# Scalable 3D printing of aperiodic cellular structures by rotational stacking of integral image formation

**DOI:** 10.1126/sciadv.abh1200

**Published:** 2021-09-17

**Authors:** Seok Kim, Jordan J. Handler, Young Tae Cho, George Barbastathis, Nicholas X. Fang

**Affiliations:** 1 Department of Mechanical Engineering, Massachusetts Institute of Technology, Cambridge, MA 02139, USA.; 2 Department of Mechanical Engineering, Changwon National University, Changwon, South Korea.; 3 Sloan School of Management, Massachusetts Institute of Technology, Cambridge, MA 02142, USA.; 4 Singapore-MIT Alliance for Research and Technology (SMART) Centre, 1 Create Way, Singapore 138602, Singapore.

## Abstract

The limitation of projection microstereolithography in additive manufacturing methods is that they typically use a single-aperture imaging configuration, which restricts their ability to produce microstructures in large volumes owing to the trade-off between image resolution and image field area. Here, we propose an integral lithography based on integral image reconstruction coupled with a planar lens array. The individual microlenses maintain a high numerical aperture and are used to create digital light patterns that can expand the printable area by the number of microlenses (10^3^ to 10^4^), thereby allowing for the scalable stereolithographic fabrication of 3D features that surpass the resolution-to-area scaling limit. We extend the capability of integral lithography for programmable printing of deterministic nonperiodic structures through the rotational overlapping or stacking of multiple exposures with controlled angular offsets. This printing platform provides new possibilities for producing periodic and aperiodic microarchitectures spanning four orders of magnitude from micrometers to centimeters.

## Teaser

Multiple tiny lenses like an insect compound eye can be used to print scalable 3D aperiodic structures.

## INTRODUCTION

Rapid developments in the fabrication of three-dimensional (3D) printed architectures have revolutionized the production of functional structures for mechanical/acoustic metamaterials (*1*–*3*), cellular mechanobiological materials (*4*), and structures for energy/environmental applications (*5*, *6*). For instance, 3D microstructures with mechanically compliant materials and customized constructed scaffolds offer tailored functionality for biocompatibility and defined stiffness (*4*). Moreover, the application of functional structures in catalytic systems has improved efficiencies by using microscale and nanoscale architectures designed to increase surface area–to–volume ratios with reduced mass (*5*). Furthermore, advances in additive manufacturing techniques have allowed for the fabrication of functional structures with complex architectures at various spatial scales down to the submicrometer scale (*6*–*8*). The commonly used stereolithography technique supports the fabrication of high-resolution and geometrically complex products (*6*, *7*), and recent advances have substantially improved feature resolution (*9*, *10*), speed (*11*), and build size (*12*–*15*). For instance, digital micromirror devices (*9*) and spatial light modulators (*12*) can be used to cure large areas [termed projection microstereolithography (PμSL)], as opposed to the conventional “tracing” approach used by single- or multiple-spot laser systems (*16*). Recent works have demonstrated the variants of PμSL that incorporate a serial printing process in which many repeated scanning cycles expand the overall build size without sacrificing resolution (*12*–*15*). One recent derivative of PμSL, named volumetric printing, overcomes the current layer-by-layer manufacturing approach to fabricate 3D objects almost instantaneously (*17*–*19*).

However, despite these system improvements, conventional PμSL methods use an imaging platform that relies on a single-aperture imaging system in which an incoming image is focused directly onto a single planar area. Consequently, the amount of transferred spatial information is fundamentally limited by the space-bandwidth product (SBP) of the pixelated digital projection system. The SBP is defined as the number of pixels required to realize the maximum information capacity. The SBP of a conventional PμSL platform is typically in the megapixels range regardless of the numerical aperture (***NA***) or magnification (***M***) of imaging optics. This results in a trade-off between the achievable minimum feature size and the total image area (*7*, *20*). This trade-off must be eliminated to further advance microstructural 3D printing for use in production.

This problem can potentially be solved by using an image multiplication strategy (i.e., numbering-up) in conjunction with a planar micro-optical imaging system. With continued advances in low-cost and large-scale microlens array fabrication techniques, micro-optical devices have become a promising tool for large-area display applications such as integral imaging 3D displays (*21*). A benefit of these fabrication techniques is that they are scalable. Image multiplication via micro-optical imaging devices has been demonstrated in Talbot array illumination (*22*, *23*) and microlens projection lithography (*24*), which are capable of fabricating submicrometer 2D lattice structures. However, the use of a static photomask limits the imaging function to a simple duplication of a single object, and therefore, it does not satisfy the design requirements for complex architectures with multiple layers beyond 2D planar structures.

At present, micro-optical and single-aperture imaging systems require further development and no existing technologies can support a scalable SBP in 3D printing. In this work, we propose a new stereolithographic printing system that uses integral image formation by a planar micro-optical device to provide a scalable additive manufacturing method without requiring serial scanning. The proposed engineered projection system is based on a lens array, in which each microlens can maintain a high ***NA*** and the overall print area can be increased with the number of microlenses. The micro-optical device combined with digital light processing allows for a scalable reconstruction of projected output images via the parallel transfer, superposition, and integration of multiple incoming images, producing periodic microarchitectures spanning four orders of magnitude from the micrometer scale to centimeter scale. We also evaluate the scalability of the integral lithographic approach and its ability to increase print areas by 10^2^ to 10^3^ times compared to current commercial PμSL systems, which translates to an SBP of 0.1 to 0.28 gigapixels (Gpx). Furthermore, we demonstrate the extended printing capability of integral lithography to create aperiodic structures by harnessing the rotational stacking of multiple integral projections with controlled angular offsets.

The aperiodic structures, which are ordered without translation symmetry observed in many natural materials (*25*), can provide a large design space and abundant structural and physical properties compared to periodic ones (*26*). In particular, aperiodic structures generated by deterministic mathematical rules (deterministic aperiodic structures) have recently attracted significant attention in engineering applications such as droplet dynamics, acoustic and optical wave transport, and photonics (*27*–*35*) due to their simplicity and geometrical freedom of design. However, the tunable and scalable printing of 3D aperiodic structures remains a difficult challenge due to time-consuming and costly fabrication techniques, thereby limiting the applications of aperiodic structures. In this study, we propose an integral lithographic approach as a promising solution to enabling the scalable printing of aperiodic cellular structures.

## RESULTS

### Integral lithographic system

**Fig. 1.**
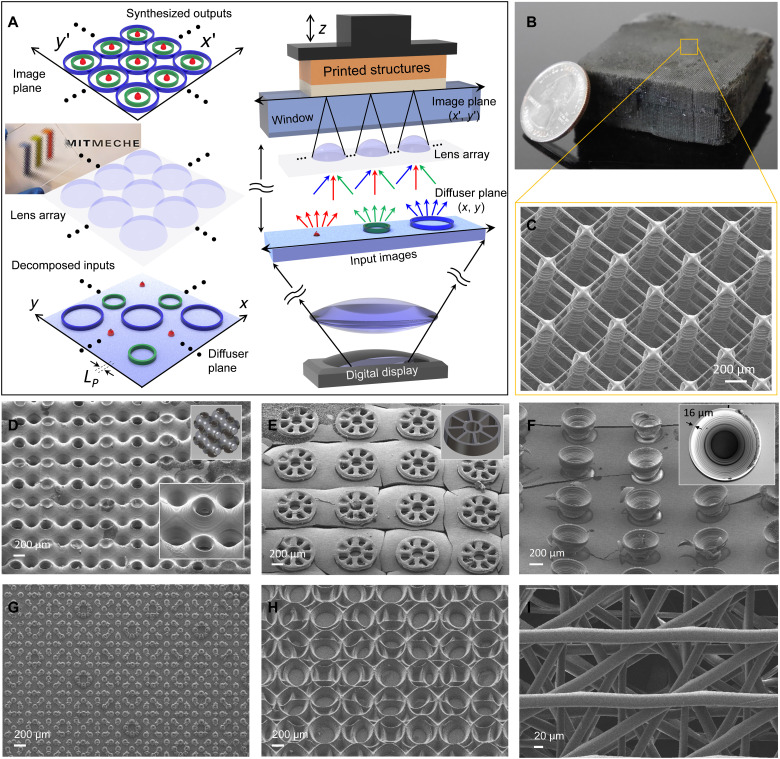
Integral lithographic system for scalable 3D printing. (**A**) Schematic of the integral lithographic system. The reconstructed imaging patterns are projected by the lens array (displayed in front of the Massachusetts Institute of Technology mechanical logo) in conjunction with the digital microdisplay. (**B** to **F**) Periodic microstructures were fabricated via linear stacking during layer-by-layer printing with an exposure time of 3 to 30 s at an intensity of 24.75 mW/cm^2^. These multiscale structures were produced by the lens array (lens 1, defined in the caption of Fig. 4 and in Materials and Methods): (B) cubic-truss microlattices (400 layers with a polymerization thickness of 5 to 50 μm), (C) scanning electron micrograph of microlattices with a strut suspended beam diameter of 5 μm; (D) triply periodic bicontinuous structures (60 layers with a polymerization thickness of 20 μm); (E) circular-lattice microscaffolds (10 layers with a polymerization thickness of 10 μm); (F) trapezoidal shell-type microstructures with a reentrant geometry (20 layers with a polymerization thickness of 20 μm). (**G** to **I**) Nonperiodic microstructures created via rotational stacking with precisely controllable angular offsets during layer-by-layer printing. These structures were fabricated by a lens array (lens 2, defined in the caption of Fig. 4 and in Materials and Methods): (G) eightfold quasi-lattices with hetero sublattices and (H) identical sublattices; (I) aperiodic woodpile lattices stacked at an angle of 2π/12 with a linear angular sequence. Photo credit: (A and B) Seok Kim, Massachusetts Institute of Technology.

A schematic overview of the proposed 3D printing system is shown in Fig. 1A. A digitally generated object image is projected onto a diffuser, which acts as the input image plane, and observed by a lens array (*24*, *36*, *37*). The lens array (displayed in Fig. 1A in front of the Massachusetts Institute of Technology MechE logo) focuses light sources from multiple viewpoints to replicate and reconstruct images into new patterns (*38*, *39*). This functionality enables incoming images to be superimposed and integrally reconstructed. The engineered projection-based printing system allows for the high-resolution and scalable stereolithographic manufacturing of complex microstructures by using the versatile imaging functions in conjunction with the lens array with the microdisplay device. During the printing process, multiple output images, each of which is generated by a unit lens of the lens array, form reconfigurable synthetic patterns via one or more combinations of replication, superposition, and integral reconstruction. Then, a set of these reconstructed images is used to create 3D architectures via linear or rotational stacking during layer-by-layer printing. The prints of the complex 3D microstructures are shown in Fig. 1 (B to I) and fig. S1. The minimum feature sizes of these microstructures are ~5 to 20 μm, and their areas are several tens of square centimeters. The microstructures demonstrate the feasibility of printing polymeric structures that exceed the resolution-to-area scaling limit. The microlattices shown in Fig. 1 (B and C) are fabricated using 400 layers of reconstructed output patterns and a polymerization layer thickness of 5 to 50 μm. The cubic-truss lattice shown in these figures is composed of three freestanding mesh layers that are suspended on an array of vertical posts and separated by identical distances in the vertical direction. These polymeric microlattices can be used at scale in customized mechanical environments, such as to mimic artificial axons (*4*) or form a catalytic reactor with a high surface area–to–volume ratio (*40*). Our approach allows for the fabrication of complex 3D microstructures that are difficult to fabricate using conventional projection lithography processes. For example, we 3D print a wide variety of structures by varying the geometric overlap of the image outputs from each unit lens. The printed structures range from interconnected bicontinuous structures (Fig. 1D) to isolated microarchitectures of circular-lattice scaffolds (Fig. 1E) and trapezoidal re-entrant structures (Fig. 1F). These examples of complex 3D microstructures with different degrees of connectivity can be extended to a variety of tissue scaffolds (*41*), mechanical metamaterials (*42*), feed spacers for water reuse system (*43*), or functional surfaces (*44*). In addition to the periodic microstructures in Fig. 1 (B to F), our approach allows for the digital printing of aperiodic microstructures based on broken lattice-dependent symmetry (Fig. 1, G to I) with different degrees of periodicity. Aperiodic microstructures can be used to create exotic metasurfaces or woodpile structures for wave engineering (*27*–*29*).

**Fig. 2.**
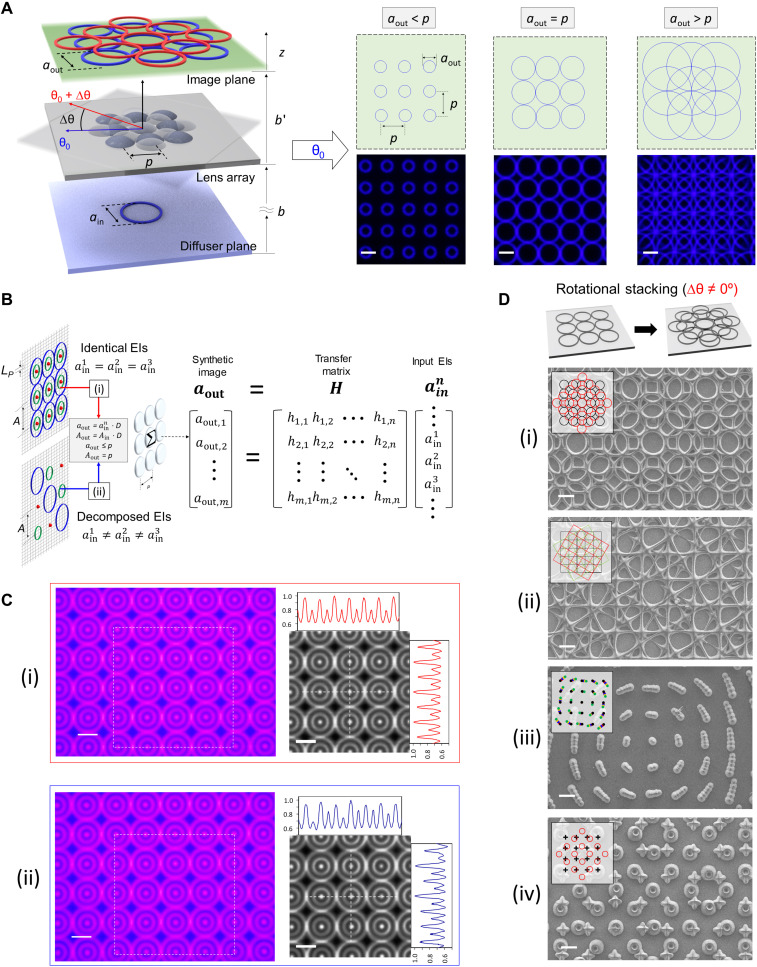
Digitally controlled imaging patterns. (**A**) The geometric relationship between the lens array and an input object produces kaleidoscopic interwoven patterns. The synthetic images are projected on an imaging plane of the lens array (lens 2) and captured by an optical microscope. Parallel replication of a single object image by the lens array, which captures an object image and generates an array of repetitive patterns (***a***_**out**_/***p*** ≤ 1). Kaleidoscopic interwoven patterns form through the overlap and superposition of multiple replicated images based on the interaction between the lens array and a single object image (***a***_**out**_/***p*** > 1). Scale bars, 100 μm. (**B** and **C**) Integral imaging patterns with compressive multiprojection: (B) Matrix form of the integral image formation between input objects of identical/decomposed elemental images (EIs) and projected outputs. Transfer matrix ***H*** is determined by its elements, ***h***_***m*,*n***_, which represent the impulse response function of the unit lens in the lens array. ***m*** and ***n*** represent the numbers of unit lenses in the lens array in the horizontal and vertical directions, respectively; (C) optical microscope–captured topologies and cross-sectional intensity profiles of integral imaging patterns created by a digital microdisplay with a projected pixel size (***L***_***P***_) of 50 μm. The intensity profiles were normalized to the maximum gray value versus the pixel distance. (i and ii) Integral imaging patterns with the identical EIs and three decomposed EIs, respectively, of the concentric circular grating on the imaging plane through the lens array. (**D**) Aperiodic lattices with rotational symmetries via multiple integral projections. Broken lattice-dependent symmetry of (i) quasi-lattices with identical bilayer (angular offset: 45°), (ii and iii) superlattices with identical trilayer (angular offsets: ± 30°) and multilayer (angular offsets: 3°), and (iv) incommensurate Moiré lattices with hetero multilayer (angular offset: 45°). These structures were printed using lens 2. Scale bars, 100 μm.

Figure 2 shows how the imaging mechanism manipulates the projected output patterns, and Fig. 2A shows the geometric relationship between the lens array, input image, and output image. The input image information is transferred in parallel by the lens array to generate an array of repetitive patterns that can produce complex patterns beyond simple replicated images. The relationship between the input image size, ***a***_**in**_, the output image size, ***a***_**out**_, and the overlap of resulting output images is given by the simple thin-lens equation 1/***f*** = 1/***b*** + 1/***b***′ (*45*), where ***f*** is the effective focal length of the unit lens, ***b*** is the distance between the lens array and input image plane, and ***b***′ is the distance between the lens array and the output image plane. The demagnification factor of the output image from each unit lens is defined as ***D*** = ***b***′/***b*** = ***f***/(***b*** − ***f***), and the resulting ***a***_**out**_ of each unit lens is ***a***_**in**_ × ***D***, as shown in Fig. 2A. The lens array is mounted on a microtranslation stage, which allows for longitudinal movement along the *z* axis to control the ***D*** of the output image by adjusting ***b*** from the input image plane. Note that we assume that the size of the unit lens is equal to the lattice spacing, ***p***, of the lens array. When ***a***_**out**_ is larger than ***p***, the multiplied images interconnect and overlap with each other to reproduce kaleidoscopic interwoven patterns in the same imaging plane (see figs. S2 to S4 for details).

The homogeneous light distribution on the lens array from the diffuser enables images from different perspectives (i.e., not orthogonally projected) to be combined in the reconstruction process (*24*, *37*). In contrast to the patterns generated from the parallel transfer and superposition of a single-input image, as shown in Fig. 2A, these synthetic patterns are created by imaging techniques that are analogous to the integral imaging techniques used in a multiview 3D display (*36*). Each unit lens of the lens array can observe multiple elemental images (EIs) and reconstruct them into identical and/or highly periodic composited patterns, as shown in Fig. 2 (B and C). To simply describe the relationship between the elements of the input objects and output images, we assume the optical system of a 1D model with column vectors $a_{\text{in}}^{n}$ and ***a***_**out**_ and optical system matrix ***H***, where $a_{\text{in}}^{n}$ and ***a***_**out**_ are the elements of the input objects and the projected images, respectively (see Materials and Methods). Then, as shown in Fig. 2B, the system can be described as $\left[a_{\text{out}}]=[H][a_{\text{in}}^{n}\right]$ considering the matrix ***H*** adopted from the image reconstruction of the light-field imaging system (*46*–*48*) and image multiplication of Talbot array illumination (*22*, *49*). The spacing, ***A***_**in**_, of the EIs is reduced by a factor of ***D*** to form the spacing, ***A***_**out**_, of the output image array. A geometrical condition described as ***A***_**out**_ = ***p*** and ***a***_**out**_ ≤ ***p*** allows for the multiview reconstruction by the superimposed images. This enables the use of multiple subimages to create a desired repetitive composited pattern or a continuous networked pattern. Consequently, scalable projected patterns can be created in stereolithographic additive manufacturing. To prove the concept of the integral imaging patterns, Fig. 2B illustrates the synthesized imagery created by digitally interlacing a set of EIs with identical ($a_{\text{in}}^{1}=a_{\text{in}}^{2}=a_{\text{in}}^{3}$) or three decomposed ($a_{\text{in}}^{1}\neq a_{\text{in}}^{2}\neq a_{\text{in}}^{3}$) spatial components. In both cases, the input objects are spatially multiplexed and decoded as synthetic images via integration in the imaging plane of the lens array. As the illumination sources are incoherent, the intensity distribution of the synthetic images from the lens array can be assumed to be a simple linear superposition of all reduced EIs. The overall surface topologies and cross-sectional intensity profiles of the projected patterns (Fig. 2C) confirm the consistency between the composite patterns created via integral imaging (see figs. S5 and S6). This integral imaging with the sparse spacing of decomposed EIs, which is termed compressive integral imaging in this study, can provide considerable benefits when coupled with inexpensive and low-bandwidth display units (see fig. S7 and the details in the Supplementary Materials). Considering frequency analysis based on a simple 1D model assumption (fig. S7A) and the Nyquist sampling criteria (***v***_**Nyq**_ = 1/2***L***_***P***_), a large display bandwidth (***L***_***P***_ of ~50 μm) can provide sufficient spatial resolution to prevent aliasing (i.e., overlapping spectral components) in integral imaging with identical and decomposed EIs (Fig. 2C). However, a low-bandwidth display unit with an ***L***_***P***_ of ~220 μm results in aliasing in the integral imaging of identical EIs (fig. S6B-i) owing to insufficient spatial resolution. Herein, a loss of intensity and slightly asymmetric profiles at the reconstructed image plane (fig. S5) are expected, but the use of compressive integral projection to decompose the high-frequency spatial component of the initial target image can provide a solution restoring the desired target image (fig. S7B-ii).

### Printing of aperiodic cellular structures through rotational overlapping/stacking of multiple integral projections

With the integral image formation, we extend the printing ability of integral lithography to programmable fabricate complex deterministic lattices with chiral or nonperiodic features through the rotational overlapping or stacking of multiple integral projections with controlled angular offsets. Along with the predictable outcomes of rotationally superimposing periodic lattices, the deterministic aperiodic structures can reproducibly create specific potential landscapes whose Fourier components are determined by the underlying aperiodic sequence. We also demonstrate sophisticated incommensurate aperiodic lattices by overlapping or stacking the periodic integral projections of different dynamic images or different angular offsets. We use the rotational stereolithographic configuration that uses an arbitrary, ***N***, of repeated exposures as a method for fabricating scalable aperiodic structures. The method is inspired by the mathematical concept of Penrose tiling (*25*), which generates quasicrystalline tilings through the superposition of distinct grids. This approach enables us to fabricate complex deterministic aperiodic lattice structures by controlling the integral imaging patterns and their angular offsets at each exposure. Figure 2D shows the representative programmable printing of aperiodic lattices including quasicrystalline lattices (quasi-lattices) with rotational symmetry (i), superlattices with nonequiangular offsets (ii and iii), and incommensurate lattices with no quasiperiodicity or superperiodicity (iv) (see more detailed results in the Supplementary Materials). Depending on the rotation angle, the printed lattices may have different aperiodic structures without translational periodicity, but they exhibit the rotational symmetry of the sublattices. In addition, the structures can transform into quasicrystals with higher rotational symmetry or aperiodic albeit regular symmetry (i.e., lattice-dependent symmetry breaking). Therefore, these structures are a promising tool for exploring the diverse physics including the investigation of liquid droplet dynamics (*30*), wave transport, and controlling the properties of wave patterns, which are relevant to several areas of acoustic metasurfaces (*27*), chiral structures (*28*), wave localization (*31*, *32*), and tunable multiband responses of quasi-lattice metasurfaces (*33*).

### Scalable photopolymerization

**Fig. 3.**
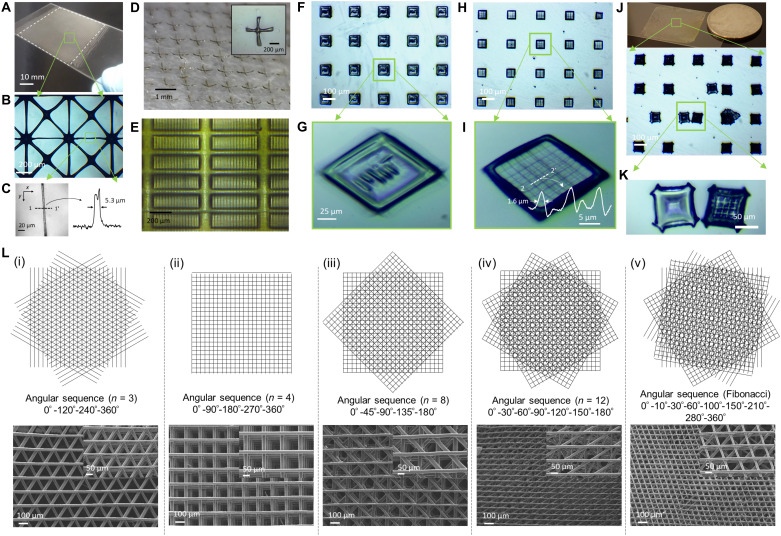
Scalable printing with small feature sizes. (**A** to **E**) Microstructures/particles created using lens 1 (***f*** = 5.5 mm, ***p*** = 1 mm, effective ***NA*** of 0.091, and an overall size of 50 × 50 mm^2^): (A to C) Periodic microstructures, such as fibrous lattice, with a minimum feature size of ~5.3 μm over an exposure area of up to 2500 mm^2^; (D) flexible multiarm microparticles; (E) microtextured surfaces. (**F** to **K**) Arbitrary micropatterns/particles fabricated using lens 3 (***f*** = 0.57 mm, ***p*** = 0.25 mm, effective ***NA*** 0.219, and an overall size of 25 × 25 mm^2^): (F to I) Array lines with feature sizes down to ~1.6 μm and array letters MiT with a maximum exposure area of up to 625 mm^2^; (J and K) 3D microparticles with a microwell array. All microstructures were printed by using the integral imaging patterns of identical EIs with a single exposure of 3 to 10 s at an intensity of 24.75 mW/cm^2^. The line profiles of the optical images shown in (C) and (I) were quantitatively analyzed using the ImageJ software. (**L**) Three-dimensional woodpile lattices with a (i to iv) linear or (v) nonlinear angular sequence forming (i and ii) periodic or (iii and v) aperiodic structures with feature sizes down to ~20.5 μm in the ***x***-***y*** plane. Depending on the rotation angle, 3D twisted woodpile lattices can be chiral structures [e.g., ***N*** = 3 for (i) and ***N*** = 8 for (iii)]. Three-dimensional woodpile structures were printed using lens 2 (***f*** = 5.2 mm, ***p*** = 0.15 mm, effective ***NA*** of 0.014, and an overall size of 10 × 10 mm^2^). Photo credit: (A, B, D, and J) Seok Kim, Massachusetts Institute of Technology.

The coupling of digitally controlled integral imaging patterns with a lens array allows for the scalable microprinting of various structures. Intertwined fibrous lattice microstructures are printed using lens 1 with a minimum feature size of ~5 μm over an exposure area of up to 2500 mm^2^ (Fig. 3, A to C, and fig. S1, E to H). Arbitrary patterns composed of array lines (Fig. 3, F to K) with feature sizes down to 1 to 2 μm and the array letters of “MiT” with a length of 50 μm are fabricated using lens 3 (defined in the caption of Fig. 3 and Materials and Methods). Considering an exposure area of several square millimeters and a lateral feature size similar to that of the single-aperture imaging–based PμSL configuration (*9*, *10*), the areal ratio (~10^2^) of printing scales demonstrates that this imaging approach can be scaled without reducing optical resolution. Furthermore, the proposed integral lithography technique provides new opportunities in applications that require the high-throughput fabrication of custom-shaped microparticles or microtextured surfaces. For example, flexible multiarm particles (Fig. 3D), micro-wavy patterned surfaces (Fig. 3E), or 3D microparticles with microwell arrays (Fig. 3, J and K) can be fabricated to serve as customized microstructural platforms for efficient cell capture in the detection and characterization of circulating cells (*50*). In particular, the integral stereolithographic approach combined with rotational layer-by-layer stacking will be suitable for the scalable fabrication of a distinct class of 3D woodpile lattice structures for Weyl phononic structures (*34*) or chiral structures (*35*). Because of the strong geometrical correlation between microscale lattices and rotational displacements, microscale geometries can be predicted in structures generated through the 3D rotational stacking of multiple periodic lattices. Figure 3L illustrates the projection view of 3D woodpile lattices printed using lens 2 via the rotational stacking of parallel rods with different angular offset sequences. Each layer is rotated by an angle of 2π/***N*** with a linear angular sequence (Fig. 3L, i to iv) or a nonlinear angular sequence (Fig. 3L, v), and periodic (Fig. 3L, i and ii) or aperiodic lattices (Fig. 3L, iii to v) are formed in the ***x***-***y*** plane. Depending on the rotation angle, 3D woodpile lattices can be chiral structures (e.g., ***N*** = 3 for Fig. 3L, i, and ***N*** = 8 for Fig. 3L, iii). Moreover, 3D twisted woodpile lattices can lead to deterministic aperiodic structures with broken lattice-dependent symmetry in the ***x***-***y*** plane (Fig. 3L, iii to v). The deterministic aperiodic lattices can provide exciting opportunities in studying transport mechanisms such as wave localization phenomena (*29*). On the basis of the predictable features by the interlayer rotation in superimposing of periodic lattices, we expect to observe new unexplored phenomena such as the exotic lattices of chiral or nonperiodic features.

## DISCUSSION

**Fig. 4.**
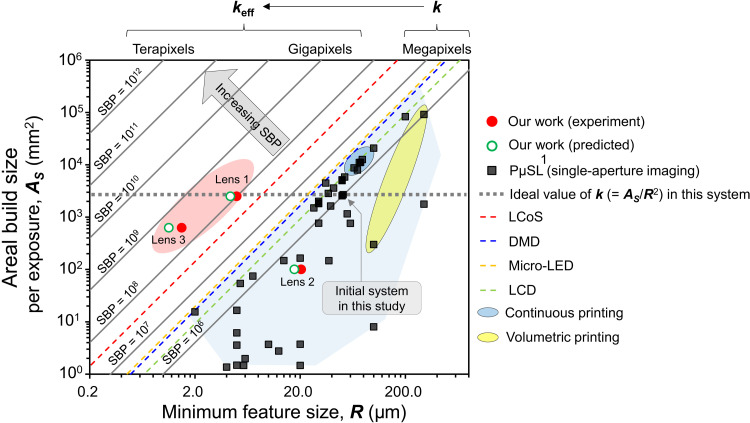
Figure of merit for the integral lithographic system. Comparison of the PμSL methods as a function of the areal build size (***A***_***S***_) versus the achievable minimum feature size (***R***). The dashed line represents analytical scaling equations grouped by the following digital microdisplay devices: Liquid crystal on silicon (LCoS) (*53*), DMD (*49*), micro–light-emitting diode (LED) (*54*), or liquid crystal display (LCD) (*55*). The data points are plotted using the published results for PμSL (based on a single-aperture imaging system) that exhibit an empirical scaling dependency. The comprehensive dataset used to produce the plot is provided in table S1. The red dots and green circles represent the experimental and the calculation results obtained by the authors, respectively, to determine the potential of integral lithography. Continuous printing (*11*); volumetric printing (*17*, *18*).

In single-aperture imaging systems based on a pixelated digital microdisplay (*51*), the areal build size (***A***_***S***_) during unit exposure is defined as (total pixels) ∙ (***L***_***D***_/***M***)^2^ (in square millimeters), where ***L***_***D***_ is the display pixel size of the constituent digital microdisplay and ***L***_***D***_/***M*** is equal to ***L***_***P***_. A rational strategy for reducing the feature size ***L***_***D***_ is to increase ***M***. However, reducing ***L***_***D***_ by using a ×10 magnification lens (i.e., ***M*** = 10) will decease ***A***_***S***_ by 100 times. Thus, the scaling problem of increasing ***A***_***S***_ without decreasing feature size remains a challenge in PμSL. To investigate the effect of the integral lithographic system on the scaling issue, we analyzed ***A***_***S***_ and the minimum feature size (***R***) for a range of existing PμSL products with available digital microdisplay devices. On the ***A***_***S***_-***R*** plot shown in Fig. 4, ***R*** is rendered as (***L***_***D***_/***M***) (*52*, *53*). The figure also shows the scaling limit, which is the ability of existing projection-based 3D printing technologies to scale microstructures. The empirical scaling behavior is deduced from the published specifications of PμSL machines (gray square dots in Fig. 4). The relationship ***A***_***S***_ = ***k*** ∙ ***R***^2^ is obtained based on theoretical analysis by following the apparent scaling dependence of the PμSL approach. ***k*** is the scaling constant corresponding to the total pixels within available digital microdisplay devices (*49*, *53*–*55*), and it refers to the SBP in the optical imaging system. In Fig. 4, these analytic scaling boundaries are denoted by dashed lines, where the red and green circles represent the experimental and calculation results obtained for the proposed printing system, respectively. The scaling constant for the relationship between the areal build size (***A***_***I***_) of integral lithography and the minimum feature size is different from the scaling constant for the ***A***_***S***_-***R*** relationship for PμSL. On the basis of the empirical illumination distribution in our system, the achievable maximum condition can be described as ***A***_***I***_ ≤ ***A***_***S***_ because the uniform illumination region and its resulting ***A***_***S***_ are determined by the maximum area of the virtual imaging mask to be observed by the lens array (*56*). Considering this condition, we estimate the ***A***_***S***_-***R*** relationship for integral lithography to compare its performance with that of PμSL as shown in Fig. 4. The effective minimum feature size, ***R***_**eff**_, of the lens array is assumed as ***R*** × ***D*** by considering geometric optics. The corresponding equation can be interpreted as

$$A_{I}\leq A_{S}=k∙R^{2}=k∙{\left(\frac{R_{\text{eff}}}{D}\right)}^{2}=(\frac{k}{D^{2}})∙{R_{\text{eff}}}^{2}=k_{\text{eff}}∙{R_{\text{eff}}}^{2}$$ (1)

where ***k***_**eff**_ is ***k***/***D***^2^ and ***R***_**eff**_ must be compliant with the Abbe diffraction-limited spot size, ***d*** = 1.22**λ**/2***NA*** (*57*), where the ***NA*** of the unit lens is defined by ***p***/2***f*** (*58*, *59*). All printing experiments are performed at an imaging distance, ***b***, of 68.75 mm. In addition, the demagnification factors, ***D***, for lens 1, lens 2, and lens 3 are 0.087, 0.082, and 0.0084, respectively, after considering the geometric condition of the lens array. The ideal ***k***_**eff**_ can be defined as ***A***_***S***_/***R***_**eff**_^2^ according to Eq. 1, plotting with a gray-colored dashed line in Fig. 4. Considering the available size of each lens array, the predicted ***k***_**eff**_ for lens 1, lens 2, and lens 3 is calculated as 1.35 × 10^8^ (~0.14 Gpx), 3.23 × 10^5^ (~0.32 Kpx), and 5.17 × 10^8^ (~0.52 Gpx), respectively (the details are provided in Materials and Methods). Furthermore, we obtain an experimental ***k***_**eff**_ of 1 × 10^8^ (0.1 Gpx), 2.38 × 10^5^ (~0.24 Kpx), and 2.78 × 10^8^ (~0. 28 Gpx) for lens 1, lens 2, and lens 3, respectively, based on the printed results. As marked on the upper left side of the lines that represent the theoretical scaling plot in Fig. 4, our approach demonstrates the potential to overcome the conventional scaling behaviors of the ***A***_***S***_-***R*** relationship (SBP-***R*** plot is shown in fig. S8). The discrepancy between the ideal ***k***_**eff**_ and predicted or experimental ***k***_**eff**_ is attributed to the limit of ***A***_***I***_ depending on the available size of the lens array and digital microdisplay devices. In addition, the obtainable minimum feature size, ***R***_**eff**_, is determined by overall contribution from photopolymerization kinetics (*9*) and the performance of the imaging system (e.g., the effective ***NA*** of the available lens array). Accordingly, we believe that these discrepancies do not imply a fundamental limit in the performance of this approach. An analysis of material deposition rate and minimum feature size of the present system to existing polymer-based 3D printing methods is also present in fig. S9, showing that this approach is efficient and competitive with commercial systems. The loss of intensity by the diffuser also limits the deposition rate of the present system, and this limit can be readily circumvented by combining a high-power illumination module.

In its present implementation, although the geometries that can be fabricated are complex or aperiodic, the main limitation to the integral lithographic approach arises from the repetitive nature of projected imaging patterns that are not fully arbitrary. The integral projection of only three identical or decomposed EIs was used to create the desired target image in this study; however, we expect that further computer-aided optimization might be obtained by addressing the inverse problem of integral image reconstruction (*60*) and that this could slightly mitigate the fundamental constraints due to the repetitive unit. As another consideration of this approach is the nonidealities in integral image patterns such as geometrical aberration or vignetting. These are challenges that need to be addressed, both to achieve the necessary uniformity for generating desired geometric features and to ensure good printing quality. Advanced lens array technologies [e.g., the aspheric lens design (*61*) or immersion method (*62*) and the computational correction procedure by inverse image function obtained after measuring nonuniformity of intensity distribution (*12*, *63*, *64*)] would be possible approaches to the aberration or vignetting issue, but further investigation in this area should be required. Furthermore, we expect that the integral lithographic approach would expand their potentials and flexibility via combining with the functional lens array such as tunable lens (*65*), multifocal lens (*66*), or various arrangements of lens array (e.g., hexagonal or nonperiodic) (*24*, *67*).

In summary, we envision that the proposed approach will be used to expand the capabilities of fabricating periodic or deterministic aperiodic microstructures with large areas and mechanical and structural benefits that are yet to be fully used at practical scales in volume production applications. If such microarchitectures are made accessible at scales larger than those that currently exist, architected materials, such as those described here, could have widespread applications, e.g., biomedical devices (*4*), extraordinary mechanical systems (*42*), functional textured surfaces (*44*), substrates for energy conversion systems (*40*, *43*), and metastructures for wave engineering (*28*, *29*, *31*–*35*). Moreover, our integral lithographic system could be incorporated into other digital light processing–based lithography systems with different types and sizes of display systems to increase the build areas of the systems further using simple and inexpensive components. This compatibility may motivate the integration of our approach with digital optofluidic fabrication for high-throughput microparticle synthesis (*68*). Our work not only provides a scalable stereolithographic microfabrication platform for periodic or deterministic aperiodic printing but also provides new possibilities for the mass production or large-scale fabrication of microstructures/particles.

## MATERIALS AND METHODS

### Printing experiment

The integral lithographic system was implemented by modifying the optical platform in a conventional PμSL system (Autodesk, Ember) composed of a digital micromirror device (DMD)-based digital microdisplay with a 405-nm light-emitting diode source (Wintech PRO4500), delivery optics, an optical diffuser (Thorlabs, DG100X100-1500), and the lens array, as shown in Fig. 1A. The initial intensity at the projection window in the PμSL system was 33 mW/cm^2^. After passing through the diffuser plane with a transmittance of ~75%, the intensity was measured at 24.75 mW/cm^2^. Note that the initial conditions of ***R*** and ***A***_***S***_ for the PμSL machine used in this work were ~50 μm and 2.56 × 10^3^ mm^2^, respectively. According to the relationship ***A***_***S***_ = ***k*** ∙ ***R***^2^, ***k*** was calculated to be 1.02 × 10^6^. We used three types of lens arrays with different focal lengths and larger overall sizes to investigate the scalable integral lithography process. These lenses were denoted as lens 1 (RPC Photonics, MLA-S1000-f5.5; ***f*** = 5.5 mm, ***p*** = 1 mm, effective ***NA*** of 0.091, and an overall size of 50 × 50 mm^2^), lens 2 (Thorlabs, MLA150-5C; ***f*** = 5.2 mm, ***p*** = 0.15 mm, effective ***NA*** of 0.014, and an overall size of 10 × 10 mm^2^), and lens 3 [Flexible Optical B.V., APO-P(GB)-P250-F0.57; ***f*** = 0.57 mm, ***p*** = 0.25 mm, effective ***NA*** of 0.219, and an overall size of 25 × 25 mm^2^]. The lens array was mounted on a motorized rotation stage (Thorlabs, PRM1Z8) combined with a manual three-axis translation stage with 25-μm translation resolution, which allows for fine longitudinal adjustment along the *z* axis and control of the angular offsets (fig. S10). We also mounted the diffuser using a manual three-axis translation stage with 25-μm translation resolution. For lenses 1 and 2, we used a 1-mm-thick glass plate covered with optically clear fluorinated ethylene propylene (FEP) with a thickness of 50 μm (CS Hyde, Lake Villa, IL, USA) for a separation layer of the printed layers. For lens 3 particularly, the thick glass plate was replaced with a thin glass plate with a thickness of 80 to 115 μm (Thorlabs, CG00K1-Cover Glasses, #0 thickness) considering the short focal length. The predicted ***k***_**eff**_ and ***R***_**eff**_ for lens 1 were computed to be 1.35 × 10^8^ and 4.3 μm using the relationships ***k***_**eff**_ = ***k***/***D***^2^ and ***R*** × ***D***, respectively, where ***D*** was 0.087. For lens 2, as ***R*** × ***D*** (=4.1 μm, where ***D*** was 0.082) was smaller than the Abbe diffraction-limited spot size (1.22**λ**/2***NA*** = 17.6 μm), ***R***_**eff**_ was considered to be 17.6 μm and the predicted ***k***_**eff**_ was consequently calculated to be 3.23 × 10^5^. Also in lens 3, as the effective minimum feature size of ***R*** × ***D*** (=0.42 μm, where ***D*** was 0.0084) was smaller than the Abbe diffraction-limited spot size (1.22**λ**/2***NA*** = 1.1 μm), ***R***_**eff**_ was considered to be 1.1 μm. Thus, the predicted ***k***_**eff**_ was calculated to be 5.17 × 10^8^ by applying the relationship ***k***_**eff**_ = ***A***_***s***_/***R***_**eff**_^2^. For the formation of aperiodic microstructures via rotational stacking, lens 2 was placed on a motorized rotation stage to synchronize the angular offsets of the lens array unit and dynamic input images during layer-by-layer printing. The microstructures were printed at an imaging distance, ***b***, of 68.75 mm. The photocurable material consisted of 1,6-hexanediol diacrylate (Sigma-Aldrich) with a 2% (w/w) phenylbis(2,4,6-trimethylbenzoyl)phosphine oxide (Irgacure 819, Sigma-Aldrich) initiator and a 1-phenylazo-2-naphthol (Sudan 1, Sigma-Aldrich) ultraviolet (UV) absorber. The concentration of the UV absorber varied from 0.05 to 0.7% (w/w) (fig. S11). In addition, we used commercial 3D printing resins (IC142-Investment Resin, Colorado photopolymer solutions) in our implementation of the integral lithographic fabrication system.

### Imaging

The input images shown in Fig. 2 (A and C) were created on a diffuser through the digital display of a conventional PμSL system using a DMD-based digital optical engine with an ***L***_***D***_ of 7.6 μm, ***M*** of ~1/6.5, and an ***A***_***S***_ of 2.56 × 10^3^ mm^2^. The output images created by the lens array were recorded by using a microscope digital complementary metal-oxide semiconductor (CMOS) sensor (AmScope MU500, sensor pixel width of 2.2 μm) with a 2× reduction lens. For the images shown in Fig. 2 (A and C), we placed lens 2 at an imaging distance of ***b*** = 68.75 mm from the masking plane in our system. The kaleidoscopic interwoven patterns in Fig. 2A were produced by adjusting the projection image shapes and sizes from 0.92 to 3.66 mm with a ***D*** of 0.082. The focal plane of the digital microscope camera coincided with the imaging plane of the lens array (***z*** = ***b***′). We arranged identical or decomposed EIs (9 × 9) of the concentric circular grating at a distance, ***A***, of 1.83 mm to characterize the projected patterns shown in Fig. 2C (see details in the Supplementary Materials). To check the imaging quality of the integral lithographic system, we determined and interpreted the point spread function (PSF) and modulation transfer function (MTF) through lens 2 as a quantitative measure of the resolution and line pattern contrast limits (fig. S12). We exposed 1-pixel illumination (~50 μm) of the current digital light processing (DLP) projector as an image of the point source to individual unit lenses. We then captured the unit lens’ PSF through high index media (PTFE-coated glass window) using a custom-built microscope composed of a 4× objective and digital CMOS sensor (AmScope MU500). We obtained the theoretical and measured MTF by Fourier transforming the theoretical and measured PSF, respectively. The MTF at given spatial frequencies can be experimentally measured as the contrast ***C*** [defined as (***I***_max_ – ***I***_min_)/(***I***_max_ + ***I***_min_)] of the sinusoidal grating of the specified spatial frequency through the optical system, where ***I***_max_ and ***I***_min_ are the maximal and minimal intensity values of the image of the line grating, respectively. We measured the MTF of the integral lithographic system by projecting line gratings of three different spatial frequencies on lens 2 in different imaging modes such as parallel replication of a single object image and integral imaging patterns with three decomposed and identical EIs (fig. S13).

### Deterministic aperiodic lattices

The deterministic aperiodic lattices with higher complexity are inspired by Moiré patterns and can be categorized as quasi-lattices, superlattices, and incommensurate lattices. The aperiodic lattices would be classified as high-symmetry quasi-lattices when a number of identical lattices of equal pitch are superimposed with equiangular offsets. Because of the equiangular offset superposition of equal pitch lattices, the resultant reciprocal lattice has reciprocal vectors of equal magnitude and with an equal angle between them. An example of quasi-lattices is shown in Fig. 2D (i), where two square lattices with circular gratings are superimposed at equiangular offsets of 45° to create eightfold symmetric quasi-lattices. Here, the equiangular offsets of square lattices are 45°, 90°, 180°, and 360°. Superlattices are created when multiple lattices of the same pitch but nonequiangular offsets are overlapped. Examples of superlattices are shown in Fig. 2D (ii and iii), where multiple square lattices with identical structures are superimposed at nonequiangular offsets of ±30° and 3° to create 12-fold and higher-order superlattices. Rotational overlapping of hetero-multilayer in Fig. 2D (iv) can also create more sophisticated patterns with no quasiperiodicity or superperiodicity, which can be classified as incommensurate moiré lattices.

### Illumination scheme

Increasing the illumination distribution over the lens array is an important factor in achieving scalable photopolymerization. Integral imaging is particularly beneficial for large-area printing because multiple superimposed array objects increase the area of uniform illuminance, as compared to the smaller region illuminated by a single object. In the proposed configuration, a digital microdisplay device projected dynamic images onto an optical diffuser, which functioned as a virtual and reconfigurable photomask. Then, the diffuser scattered the light to produce a near Lambertian profile, which ensured homogeneous illumination in all directions in the lens array plane (*36*, *38*, *39*). The scattered light entered the lens array that is positioned at an imaging distance ***b***. Each lens in the array refocused the light to reduce the size of the images generated by the optical diffuser. The illumination distribution incident on the lens array was investigated using various object image configurations (see figs. S14 to 16). For simplicity, we used a circular shape as the virtual input image and assumed that the optical diffuser was an imperfect Lambertian emitter (see figs. S14 to S16). This simplification enabled us to use an adapted form of radiometric analysis (see figs. S14 to S16) when comparing the illumination distributions of a single object and an array of objects. On the basis of these assumptions, we derived the approximated equations of illumination distributions for a single object and an array of objects via radiometric analysis using Cartesian coordinates (the details are provided in the Supplementary Materials). The calculated and measured illumination distributions for our imaging system are shown in fig. S14. The illumination distribution was measured without the lens array using a home-built scanner (XY-axis stepping motors), which included an optical powermeter and sensor (Thorlabs; PM100D and S120VC, respectively). To reproduce an illumination environment in which the light was incident immediately below the lens array, the optical power distribution was measured over an area of 50 × 50 mm^2^ and at a step size of 0.5 mm at an imaging distance of 68.75 mm from the projected images (the details are provided in the Supplementary Materials). The measured results were plotted in the form of a 2D illumination distribution using MATLAB. The illumination distribution of a single circular source exhibited a narrow flat region, which provided limited options for scalability. However, the illumination homogeneity was notably improved by superimposing array object sources. For example, the sum of the illumination distributions for a square array of 5 × 3 circular sources is depicted in fig. S14 (D to F). The illumination distribution was uniform along the horizontal direction at imaging distance ***b*** from the diffuser. These results indicate that this illumination superposition scheme, along with the integral imaging method, can be used to generate a large-scale and uniform illumination distribution.

## Supplementary Materials

### This PDF file includes:

Supplementary Text Figs. S1 to S16 Table S1 References

## References

[1] T. A. Schaedler, A. J. Jacobsen, A. Torrents, A. E. Sorensen, J. Lian, J. R. Greer, L. Valdevit, W. B. Carter, Ultralight metallic microlattices. Science 334, 962–965 (2011). 22096194 10.1126/science.1211649

[2] X. Zheng, H. Lee, T. H. Weisgraber, M. Shusteff, J. DeOtte, E. B. Duoss, J. D. Kuntz, M. M. Biener, Q. Ge, J. A. Jackson, S. O. Kucheyev, N. X. Fang, C. M. Spadaccini, Ultralight, ultrastiff mechanical metamaterials. Science 344, 1373–1377 (2014). 24948733 10.1126/science.1252291

[3] K. Yu, N. X. Fang, G. Huang, Q. Wang, Magnetoactive acoustic metamaterials. Adv. Mater. 30, 1706348 (2018). 10.1002/adma.201706348 29638017

[4] D. Espinosa-Hoyos, A. Jagielska, K. A. Homan, H. Du, T. Busbee, D. G. Anderson, N. X. Fang, J. A. Lewis, K. J. Van Vliet, Engineered 3D-printed artificial axons. Sci. Rep. 8, 478 (2018). 29323240 10.1038/s41598-017-18744-6 PMC5765144

[5] X. Zhou, C. Liu, Three-dimensional printing for catalytic applications: Current status and perspectives. Adv. Funct. Mater. 27, 1701134 (2017).

[6] C. M. Soukoulis, M. Wegener, Past achievements and future challenges in the development of three-dimensional photonic metamaterials. Nat. Photon 5, 523–530 (2011).

[7] R. L. Truby, A. Jennifer, J. A. Lewis, Printing soft matter in three dimensions. Nature 540, 371–378 (2016). 27974748 10.1038/nature21003

[8] Y. Zhang, F. Zhang, Z. Yan, Q. Ma, X. Li, Y. Huang, J. A. Rogers, Printing, folding and assembly methods for forming 3D mesostructures in advanced materials. Nat. Rev. Mater. 2, 17019 (2017).

[9] C. Sun, N. Fang, D. M. Wu, X. Zhang, Projection micro-stereolithography using digital micro-mirror dynamic mask. Sens. Actuators A 121, 113–120 (2005).

[10] R. Raman, B. Bhaduri, M. Mir, A. Shkumatov, M. K. Lee, G. Popescu, H. Kong, R. Bashir, High-resolution projection microstereolithography for patterning of neovasculature. Adv. Healthc. Mater. 5, 610–619 (2016). 26696464 10.1002/adhm.201500721

[11] J. R. Tumbleston, D. Shirvanyants, N. Ermoshkin, R. Janusziewicz, A. R. Johnson, D. Kelly, K. Chen, R. Pinschmidt, J. P. Rolland, A. Ermoshkin, E. T. Samulski, J. M. DeSimone, Continuous liquid interface production of 3D objects. Science 347, 1349–1352 (2015). 25780246 10.1126/science.aaa2397

[12] X. Zheng, J. Deotte, M. P. Alonso, G. R. Farquar, T. H. Weisgraber, S. Gemberling, H. Lee, N. X. Fang, C. M. Spadaccini, Design and optimization of a light-emitting diode projection micro-stereolithography three-dimensional manufacturing system. Rev. Sci. Instrum. 83, 125001 (2012). 23278017 10.1063/1.4769050

[13] M. M. Emamia, F. Barazandeha, F. Yaghmaieba, Scanning-projection based stereolithography: Method and structure. Sens. Actuat. A 218, 116–124 (2014).

[14] X. Zheng, W. Smith, J. Jackson, B. Moran, H. Cui, D. Chen, J. Ye, N. X. Fang, N. Rodriguez, T. Weisgraber, C. M. Spadaccini, Multiscale metallic metamaterials. Nat. Mater. 15, 1100–1106 (2016). 27429209 10.1038/nmat4694

[15] M. P. Lee, G. J. T. Cooper, T. Hinkley, G. M. Gibson, M. J. Padgett, L. Cronin, Development of a 3D printer using scanning projection stereolithography. Sci. Rep. 5, 9875 (2015). 25906401 10.1038/srep09875 PMC4407727

[16] J. Kato, N. Takeyasu, Y. Adachi, H. Sun, S. Kawata, Multiple-spot parallel processing for laser micronanofabrication. Appl. Phys. Lett. 86, 044102 (2005).

[17] M. Shusteff, A. E. M. Browar, B. E. Kelly, J. Henriksson, T. H. Weisgraber, R. M. Panas, N. X. Fang, C. M. Spadaccini, One-step volumetric additive manufacturing of complex polymer structures. Sci. Adv. 3, eaao5496 (2017). 29230437 10.1126/sciadv.aao5496 PMC5724355

[18] B. E. Kelly, I. Bhattacharya, H. Heidari, M. Shusteff, C. M. Spadaccini, H. K. Taylor, Volumetric additive manufacturing via tomographic reconstruction. Science 363, 1075–1079 (2019). 30705152 10.1126/science.aau7114

[19] M. Regehly, Y. Garmshausen, M. Reuter, N. F. König, E. Israel, D. P. Kelly, C.-Y. Chou, K. Koch, B. Asfari, S. Hecht, Xolography for linear volumetric 3D printing. Nature 588, 620–624 (2020). 33361791 10.1038/s41586-020-3029-7

[20] M. Malinauskas, A. Žukauskas, S. Hasegawa, Y. Hayasaki, V. Mizeikis, R. Buividas, S. Juodkazis, Ultrafast laser processing of materials: From science to industry. Light Sci. Appl. 5, e16133 (2016). 30167182 10.1038/lsa.2016.133 PMC5987357

[21] X. Zhou, Y. Peng, R. Peng, X. Zeng, Y. Zhang, T. Guo, Fabrication of large-scale microlens arrays based on screen printing for integral imaging 3D display. ACS Appl. Mater. Interfaces 8, 24248–24255 (2016). 27540754 10.1021/acsami.6b08278

[22] L. Stuerzebecher, T. Harzendorf, U. Vogler, U. D. Zeitner, R. Voelkel, Advanced mask aligner lithography: Fabrication of periodic patterns using pinhole array mask and Talbot effect. Opt. Express 18, 19485–19494 (2010). 20940844 10.1364/OE.18.019485

[23] A. Kolodziejczyk, Z. Jaroszewicz, R. Henao, O. Quintero, The Talbot array illuminator: Imaging properties and a new interpretation. J. Opt. A: Pure Appl. Opt. 6, 651–657 (2004).

[24] M. Gonidec, M. M. Hamedi, A. Nemiroski, L. M. Rubio, C. Torres, G. M. Whitesides, Fabrication of nonperiodic metasurfaces by microlens projection lithography. Nano Lett. 16, 4125–4132 (2016). 27244272 10.1021/acs.nanolett.6b00952

[25] R. Penrose, The role of aesthetics in pure and applied mathematical research. Bull. Inst. Math. Appl. 10, 266–271 (1974).

[26] L. Dal Negro, S. Boriskina, Deterministic aperiodic nanostructures for photonics and plasmonics applications. Laser & Photon. Rev. 6, 178–218 (2012).

[27] H. Tang, Z. Chen, N. Tang, S. Li, Y. Shen, Y. Peng, X. Zhu, J. Zang, Hollow-out patterning ultrathin acoustic metasurfaces for multifunctionalities using soft fiber/rigid bead networks. Adv. Funct. Mater. 28, 1801127 (2018).

[28] Z. Wu, Y. Zheng, Moiré chiral metamaterials. Adv. Optical Mater. 5, 1700034 (2017).

[29] M. Renner, G. von Freymann, Transverse mode localization in three-dimensional deterministic aperiodic structures. Adv. Optical Mater. 2, 226–230 (2014).

[30] E. Chen, Q. Yuan, Y.-P. Zhao, Topography-induced symmetry transition of droplets on quasi-periodically patterned surfaces. Soft Matter 14, 6198–6205 (2018). 29808212 10.1039/c8sm00591e

[31] A. C. Hladky-Hennion, J. O. Vasseur, S. Degraeve, C. Granger, M. de Billy, Acoustic wave localization in one-dimensional Fibonacci phononic structures with mirror symmetry. J. Appl. Phys. 113, 154901 (2013).

[32] P. Wang, Y. Zheng, X. Chen, C. Huang, Y. V. Kartashov, L. Torner, V. V. Konotop, F. Ye, Localization and delocalization of light in photonic moiré lattices. Nature 577, 42–46 (2020). 31853062 10.1038/s41586-019-1851-6

[33] J.-H. Han, I. Kim, J.-W. Ryu, J. Kim, J.-H. Cho, G.-S. Yim, H.-S. Park, B. Min, M. Choi, Rotationally reconfigurable metamaterials based on moiré phenomenon. Opt. Express 23, 17443–17449 (2015). 26191753 10.1364/OE.23.017443

[34] H. He, C. Qiu, L. Ye, X. Cai, X. Fan, M. Ke, F. Zhang, Z. Liu, Topological negative refraction of surface acoustic waves in a Weyl phononic crystal. Nature 560, 61–64 (2018). 30068954 10.1038/s41586-018-0367-9

[35] M. Thiel, G. von Freymann, M. Wegener, Layer-by-layer three-dimensional chiral photonic crystals. Opt. Lett. 32, 2547–2549 (2007). 17767300 10.1364/ol.32.002547

[36] J. Geng, Three-dimensional display technologies. Adv. Opt. Photon. 5, 456–535 (2013). 10.1364/AOP.5.000456 PMC4269274 25530827

[37] A. Manakov, J. F. Restrepo, O. Klehm, R. Hegedus, E. Eisemann, H.-P. Seidel, I. Ihrke, A reconfigurable camera add-on for high dynamic range, multispectral, polarization, and light-field imaging. ACM Trans. Graph. 32, 4 (2013).

[38] J. Arai, F. Okano, H. Hoshino, I. Yuyama, Gradient-index lens-array method based on real-time integral photography for three-dimensional images. Appl. Optics 37, 2034–2045 (1998). 10.1364/ao.37.002034 18273122

[39] X. Xiao, B. Javidi, M. Martinez-Corral, A. Stern, Advances in three-dimensional integral imaging: Sensing, display, and applications [Invited]. Appl. Optics 52, 546–560 (2013). 10.1364/AO.52.000546 23385893

[40] C. Parra-Cabrera, C. Achille, S. Kuhn, R. Ameloot, 3D printing in chemical engineering and catalytic technology: Structured catalysts, mixers and reactors. Chem. Soc. Rev. 47, 209–230 (2018). 29131228 10.1039/c7cs00631d

[41] F. P. W. Melchels, K. Bertoldi, R. Gabbrielli, A. H. Velders, J. Feijen, D. W. Grijpma, Mathematically defined tissue engineering scaffold architectures prepared by stereolithography. Biomaterials 31, 6909–6916 (2010). 20579724 10.1016/j.biomaterials.2010.05.068

[42] W. Lee, D. Kang, J. Song, J. H. Moon, D. Kim, Controlled unusual stiffness of mechanical metamaterials. Sci. Rep. 6, 20312 (2016). 26837466 10.1038/srep20312 PMC4738250

[43] N. Thomas, N. Sreedhar, O. Al-Ketan, R. Rowshan, R. K. A. Al-Rub, H. Arafat, 3D printed triply periodic minimal surfaces as spacers for enhanced heat and mass transfer in membrane distillation. Desalination 443, 256–271 (2018).

[44] R. He, S. Wang, G. Andrews, W. Shi, Y. Liu, Generation of customizable microwavy pattern through grayscale direct image lithography. Sci. Rep. 6, 21621 (2016). 26902520 10.1038/srep21621 PMC4763239

[45] L. Li, A. Y. Yil, Microfabrication on a curved surface using 3D microlens array projection. J. Micromech. Microeng. 19, 105010 (2009).

[46] J. Tanida, T. Kumagai, K. Yamada, S. Miyatake, K. Ishida, T. Morimoto, N. Kondou, D. Miyazaki, Y. Ichioka, Thin observation module by bound optics (TOMBO): Concept and experimental verification. Appl. Optics 40, 1806–1813 (2001). 10.1364/ao.40.001806 18357179

[47] H. Li, C. Guo, I. Muniraj, B. C. Schroeder, J. T. Sheridan, S. Jia, Volumetric light-field encryption at the microscopic scale. Sci. Rep. 7, 40113 (2017). 28059149 10.1038/srep40113 PMC5216341

[48] M. Broxton, L. Grosenick, S. Yang, N. Cohen, A. Andalman, K. Deisseroth, M. Levoy, Wave optics theory and 3-D deconvolution for the light field microscope. Opt. Express 21, 25418–25439 (2013). 24150383 10.1364/OE.21.025418 PMC3867103

[49] TI DLP 4K Ultra High Definition (UHD) Display Chipset: www.ti.com/lit/ml/ssnb002/ssnb002.pdf.

[50] L. N. Kim, S. Choi, J. Kim, H. Kim, S. Kwon, Single exposure fabrication and manipulation of 3D hydrogel cell microcarriers. Lab Chip 11, 48–51 (2011). 20981360 10.1039/c0lc00369g

[51] H. Kang, J. H. Park, D. Cho, A pixel based solidification model for projection based stereolithography technology. Sens. Actuat. A 178, 223–229 (2012).

[52] W. Yang, H. Yu, W. Liang, Y. Wang, L. Liu, Rapid fabrication of hydrogel microstructures using UV-induced projection printing. Micromachines 6, 1903–1913 (2015).

[53] G. Lazarev, A. Hermerschmidt, S. Krüger, S. Ostena, LCOS spatial light modulators: Trends and applications, in *Optical Imaging and Metrology: Advanced Technologies*, W. Osten, N. Reingand, Eds. (Wiley-VCH, 2012).

[54] L. Zhang, F. Ou, W. C. Chong, Y. J. Chen, Q. M. Li, Wafer-scale monolithic hybrid integration of Si-based IC and III-V epi-layers—A mass manufacturable approach for active matrix micro-LED micro-displays. J. Soc. Inf. Display 26, 137–145 (2018).

[55] Epson 3LCD, 3-chip technology: https://epson.com/For-Home/Projectors/Pro-Cinema/PowerLite-Pro-Cinema-4855WU-WUXGA-3LCD-Projector-Kit/p/V11H543120MB.

[56] R. Wu, Z. Zheng, H. Li, X. Liu, Optimization design of irradiance array for LED uniform rectangular illumination. Appl. Optics 51, 2257–2263 (2012). 10.1364/AO.51.002257 22614399

[57] A. Waldbaur, B. Waterkotte, K. Schmitz, B. E. Rapp, Maskless projection lithography for the fast and flexible generation of grayscale protein patterns. Small 8, 1570–1578 (2012). 22411542 10.1002/smll.201102163

[58] S. Surdo, R. Carzino, A. Diaspro, M. Duocastella, Single-shot laser additive manufacturing of high fill-factor microlens arrays. Adv. Opt. Mater. 6, 1701190 (2018).

[59] D. Zhang, Q. Xu, C. Fang, K. Wang, X. Wang, S. Zhuang, B. Dai, Fabrication of a microlens array with controlled curvature by thermally curving photosensitive gel film beneath microholes. ACS Appl. Mater. Interfaces 9, 16604–16609 (2017). 28452461 10.1021/acsami.7b00766

[60] G. Barbastathis, A. Ozcan, G. Situ, On the use of deep learning for computational imaging. Optica 6, 921–943 (2019).

[61] D. Wu, S.-Z. Wu, L.-G. Niu, Q.-D. Chen, R. Wang, J.-F. Song, H.-H. Fang, H.-B. Sun, High numerical aperture microlens arrays of close packing. Appl. Phys. Lett. 97, 031109 (2010).

[62] A. Orth, K. Crozier, Gigapixel fluorescence microscopy with a water immersion microlens array. Opt. Express 21, 2361–2368 (2013). 23389216 10.1364/OE.21.002361

[63] M. Shusteff, “Volumetric additive manufacturing of polymer structures by holographically projected light fields”, thesis, Massachusetts Institute of Technology, Cambridge, MA (2017).

[64] Z. Xiong, H. Liu, R. Chen, J. Xu, Q. Li, J. Li, W. Zhang, Illumination uniformity improvement in digital micromirror device based scanning photolithography system. Opt. Express 26, 18597–18607 (2018). 30114036 10.1364/OE.26.018597

[65] J. Kim, Y. Jeong, H. Kim, C.-K. Lee, B. Lee, J. Hong, Y. Kim, Y. Hong, S.-D. Lee, B. Lee, F-number matching method in light field microscopy using an elastic micro lens array. Opt. Lett. 41, 2751–2754 (2016). 27304280 10.1364/OL.41.002751

[66] S.-I. Bae, K. Kim, S. Yang, K.-W. Jang, K.-H. Jeong, Multifocal microlens arrays using multilayer photolithography. Opt. Express 28, 9082–9088 (2020). 32225521 10.1364/OE.388921

[67] H. Yabu, M. Shimomura, Simple fabrication of micro lens arrays. Langmuir 21, 1709–1711 (2005). 15723463 10.1021/la046996z

[68] G. C. L. Goff, J. Lee, A. Gupta, W. A. Hill, P. S. Doyle, High-throughput contact flow lithography. Adv. Sci. 2, 1500149 (2015). 10.1002/advs.201500149 PMC5115321 27980910

[69] H. Zhang, F. Yang, J. Dong, L. Du, C. Wang, J. Zhang, C. F. Guo, Q. Liu, Kaleidoscopic imaging patterns of complex structures fabricated by laser-induced deformation. Nat. Commun. 7, 13743 (2016). 27910852 10.1038/ncomms13743 PMC5476795

[70] S. Kim, D. H. Kim, W. Kim, Y. T. Cho, N. X. Fang, Additive manufacturing of functional microarchitected reactors for energy, environmental, and biological applications. Int. J. of Precis. Eng. and Manuf.-Green Tech. 8, 303–326 (2021).

[71] E. Behroodi, H. Latifi, F. Najafi, A compact LED-based projection microstereolithography for producing 3D microstructures. Sci. Rep. 9, 19692 (2019). 31873101 10.1038/s41598-019-56044-3 PMC6928235

[72] C.-C. Sun, W.-T. Chien, I. Moreno, C.-T. Hsieh, M.-C. Lin, S.-L. Hsiao, X.-H. Lee, Calculating model of light transmission efficiency of diffusers attached to a lighting cavity. Opt. Express 18, 6137–6148 (2010). 20389636 10.1364/OE.18.006137

[73] I. Moreno, M. Avendaño-Alejo, R. I. Tzonchev, Designing light-emitting diode arrays for uniform near-field irradiance. Appl. Optics 45, 2265–2272 (2006). 10.1364/ao.45.002265 16607994

[74] H. Chen, C. Liou, S. Siao, Illumination distribution and signal transmission for indoor visible light communication with different light-emitting diode arrays and pre-equality circuits. Opt. Eng. 54, 115106 (2015).

[75] L. Han, G. Mapili, S. Chen, K. Roy, Projection microfabrication of three-dimensional scaffolds for tissue engineering. J. Manuf. Sci. Eng. 130, 021005 (2008).

[76] C. Xia, N. X. Fang, 3D micro fabricated bioreactor with capillaries. Biomed. Microdevices 11, 1309–1315 (2009). 19806459 10.1007/s10544-009-9350-4

[77] J. Choi, R. Wicker, S. Lee, K. Choi, C. Ha, I. Chung, Fabrication of 3D biocompatible/biodegradable micro-scaffolds using dynamic mask projection microstereolithography. J. Mater. Process. Technol. 209, 5494–5503 (2009).

[78] J. Choi, R. B. Wicker, S. Cho, C. Ha, S. Lee, Cure depth control for complex 3D microstructure fabrication in dynamic mask projection microstereolithography. Rapid Prototyp. J. 15, 59–70 (2009).

[79] C. Zhou, Y. Chen, Z. Yang, B. Khoshnevis, Digital material fabrication using mask-image-projection-based stereolithography. Rapid Prototyp. J. 19, 153–165 (2013).

[80] A. R. Schultz, P. M. Lambert, N. A. Chartrain, D. M. Ruohoniemi, Z. Zhang, C. Jangu, M. Zhang, C. B. Williams, T. E. Long, 3D printing phosphonium ionic liquid networks with mask projection microstereolithography. ACS Macro Lett. 3, 1205–1209 (2014). 35610826 10.1021/mz5006316

[81] K. Kim, W. Zhu, X. Qu, C. Aaronson, W. R. McCall, S. Chen, D. J. Sirbuly, 3D optical printing of piezoelectric nanoparticle–polymer composite materials. ACS Nano 8, 9799–9806 (2014). 25046646 10.1021/nn503268f

[82] A. P. Zhang, X. Qu, P. Soman, K. C. Hribar, J. W. Lee, S. Chen, S. He, Rapid fabrication of complex 3D extracellular microenvironments by dynamic optical projection stereolithography. Adv. Mater. 24, 4266–4270 (2012). 22786787 10.1002/adma.201202024 PMC3789064

[83] P. Soman, B. T. D. Tobe, J. W. Lee, A. M. Winquist, I. Singec, K. S. Vecchio, E. Y. Snyder, S. Chen, Three-dimensional scaffolding to investigate neuronal derivatives of human embryonic stem cells. Biomed. Microdevices 14, 829–838 (2012). 22767243 10.1007/s10544-012-9662-7 PMC3785556

[84] Y. L. Jeyachandran, N. Meyerbröker, A. Terfort, M. Zharnikov, Maskless ultraviolet projection lithography with a biorepelling monomolecular resist. J. Phys. Chem. C 119, 494–501 (2015).

[85] Lawrence Livermore National Laboratory (LLNL), “Large-area projection micro-stereolithography (LAPμSL)” (Publication LLNL-MI-670797, LLNL, 2015; https://ipo.llnl.gov/sites/default/files/2019-09/lapusl.pdf).

[86] M. Shusteff, “Projection microstereolithography for architected materials” (Tech. Rep. LLNL-TR-666552, Lawrence Livermore National Laboratory (LLNL), 2015).

[87] R. Janusziewicz, J. R. Tumbleston, A. L. Quintanilla, S. J. Mecham, J. M. DeSimone, Layerless fabrication with continuous liquid interface production. Proc. Natl. Acad. Sci. U.S.A. 113, 11703–11708 (2016). 27671641 10.1073/pnas.1605271113 PMC5081641

[88] H. Gong, M. Beauchamp, S. Perry, A. T. Woolleyb, G. P. Nordin, Optical approach to resin formulation for 3D printed microfluidics. RSC Adv. 5, 106621–106632 (2015). 26744624 10.1039/C5RA23855B PMC4699590

[89] A. S. Limaye, D. W. Rosen, Process planning method for mask projection micro-stereolithography. Rapid Prototyp. J. 13, 76–84 (2007).

[90] Y. Jung, H. Lee, T. Park, S. Kim, S. Kwon, Programmable gradational micropatterning of functional materials using maskless lithography controlling absorption. Sci. Rep. 5, 15629 (2015). 26490360 10.1038/srep15629 PMC4615026

[91] F. Zhou, W. Cao, B. Dong, T. Reissman, W. Zhang, C. Sun, Additive manufacturing of a 3D terahertz gradient-refractive index lens. Adv. Optical Mater. 4, 1034–1040 (2016).

[92] F. Zhou, Y. Bao, W. Cao, C. T. Stuart, J. Gu, W. Zhang, C. Sun, Hiding a realistic object using a broadband terahertz invisibility cloak. Sci. Rep. 1, 78 (2011). 22355597 10.1038/srep00078 PMC3216565

[93] A. Urrios, C. Parra-Cabrera, N. Bhattacharjee, A. M. Gonzalez-Suarez, L. G. Rigat-Brugarolas, U. Nallapatti, J. Samitier, C. A. DeForest, F. Posas, J. L. Garcia-Corderob, A. Folch, 3D-printing of transparent bio-microfluidic devices in PEG-DA. Lab Chip 16, 2287–2294 (2016). 27217203 10.1039/c6lc00153j PMC4930360

[94] Q. Ge, A. H. Sakhaei, H. Lee, C. K. Dunn, N. X. Fang, M. L. Dunn, Multimaterial 4D printing with tailorable shape memory polymers. Sci. Rep. 6, 31110 (2016). 27499417 10.1038/srep31110 PMC4976324

[95] J. Na, N. P. Bende, J. Bae, C. D. Santangelob, R. C. Hayward, Grayscale gel lithography for programmed buckling of non-Euclidean hydrogel plates. Soft Matter 12, 4985–4990 (2016). 27169886 10.1039/c6sm00714g

[96] N. P. Macdonald, J. M. Cabot, P. Smejkal, R. M. Guijt, B. Paull, M. C. Breadmore, Comparing microfluidic performance of three-dimensional (3D) printing platforms. Anal. Chem. 89, 3858–3866 (2017). 28281349 10.1021/acs.analchem.7b00136

