# Supplementary material for: Scalable 3D printing of aperiodic cellular structures by rotational stacking of integral image formation
Source: Sci Adv. 2021 Sep 17;7(38):eabh1200. doi: 10.1126/sciadv.abh1200 (PMC8448457; doi:10.1126/sciadv.abh1200)
Supplement: Supplementary file 1 — Supplementary Text Figs. S1 to S16 Table S1 References [file sciadv.abh1200_sm.pdf]

Science Advances
AAAS

Supplementary Materials for

Scalable 3D printing of aperiodic cellular structures by rotational stacking of integral image formation

Seok Kim, Jordan J. Handler, Young Tae Cho, George Barbastathis, Nicholas X. Fang*

*Corresponding author. Email: nicfang@mit.edu

Published 17 September 2021, *Sci. Adv.* **7**, eabh1200 (2021)
DOI: 10.1126/sciadv.abh1200

This PDF file includes:

Supplementary Text
Figs. S1 to S16
Table S1
References

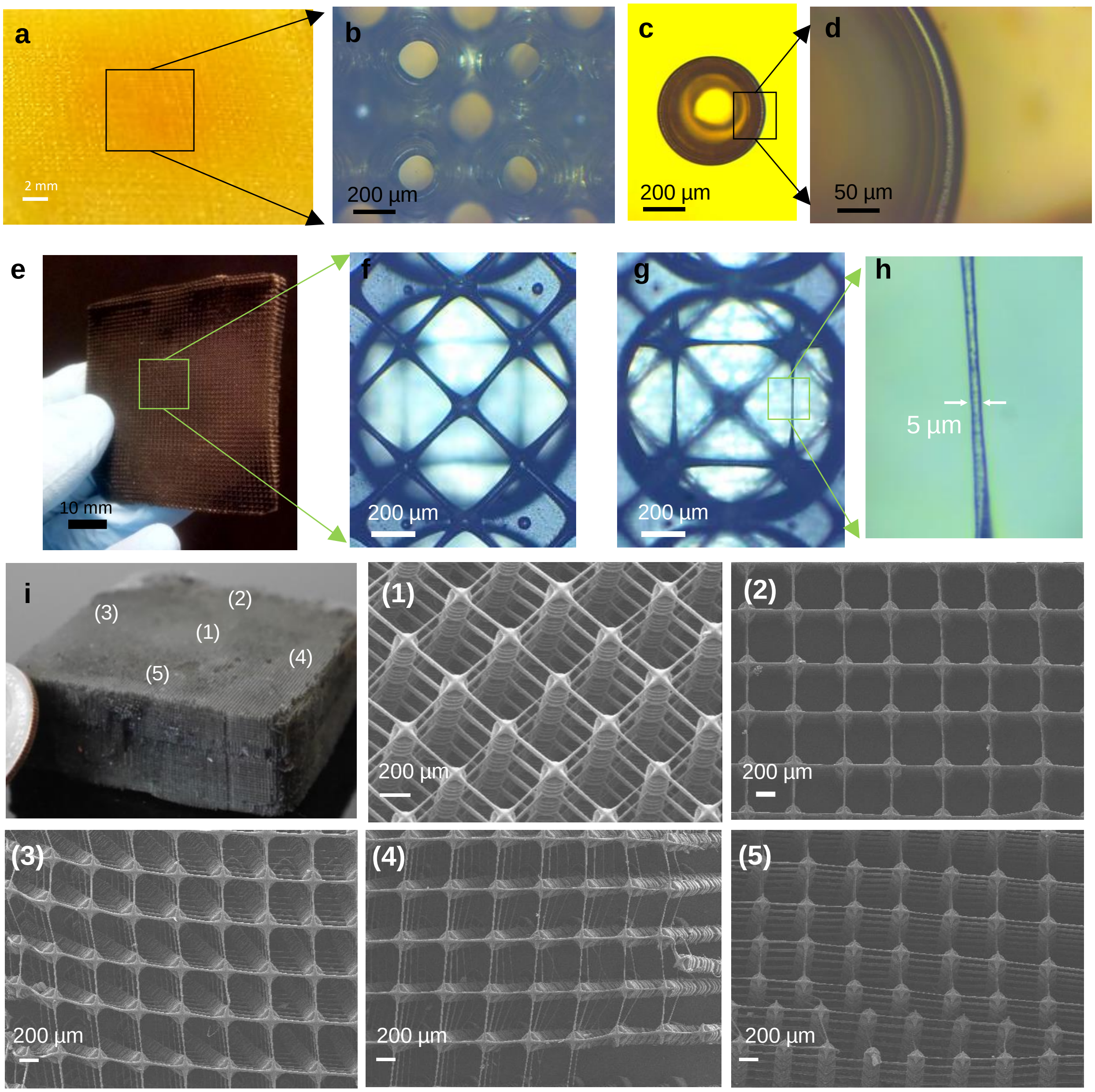

Fig. S1. Optical microscope images of 3D-printed microstructures of Fig. 1 in the main text. **(A-B)** Triply periodic bicontinuous microstructures (total layers with slicing thickness of 50 µm: 60). **(C-D)** Trapezoidal shell-type microstructures with reentrant geometry (total layers with slicing thickness of 20 µm: 20). **(E-H)** An architecture of free-standing microfiber arrays with diameters 5 - 20 µm and an overall size of ~ 50 × 50 mm^2. **(I)** Scanning electron micrograph (SEM) of cubic-truss microlattices in Fig. 1B. Photo Credit: (A, E, and I) Seok Kim, Massachusetts Institute of Technology.

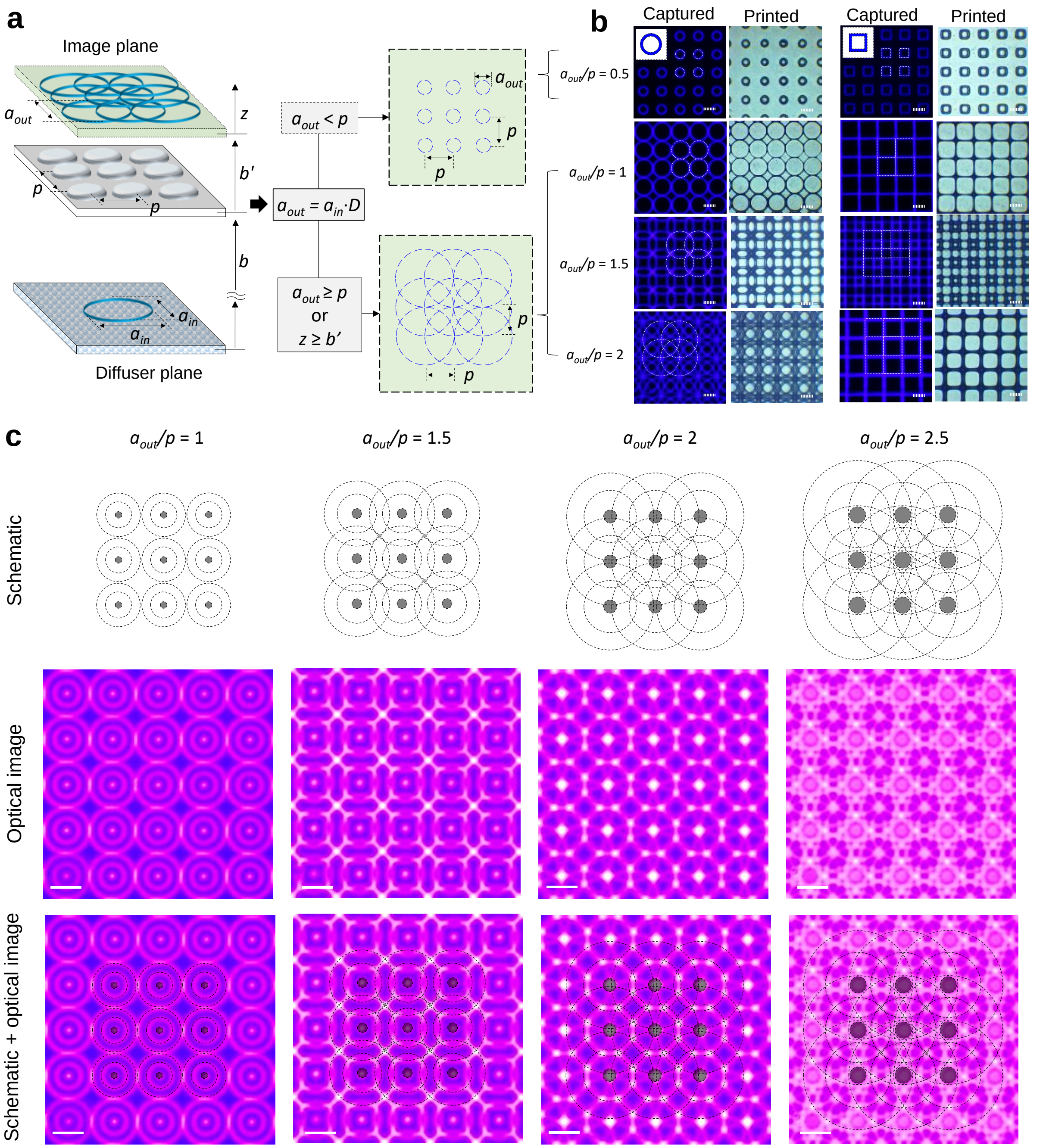

Fig. S2. Integral imaging patterns. (A) The geometric relationship between the lens array and an input object. **(B)** The captured optical microscope and printed structures via photopolymerization. Parallel replication of a single object image by the lens array, which captures an object image and generates an array of repetitive patterns ($\boldsymbol{a_{out}/p} \leq 1$). Kaleidoscopic interwoven patterns form through the overlap and superposition of multiple replicated images based on the interaction between the lens array and a single object image ($\boldsymbol{a_{out}/p} > 1$). **(C)** Kaleidoscopic patterns of a concentric circular grating with varying $\boldsymbol{a_{out}/p}$. Illustrative schematics and optical image are provided on the top and middle, respectively. All scale bars are 100 μm.

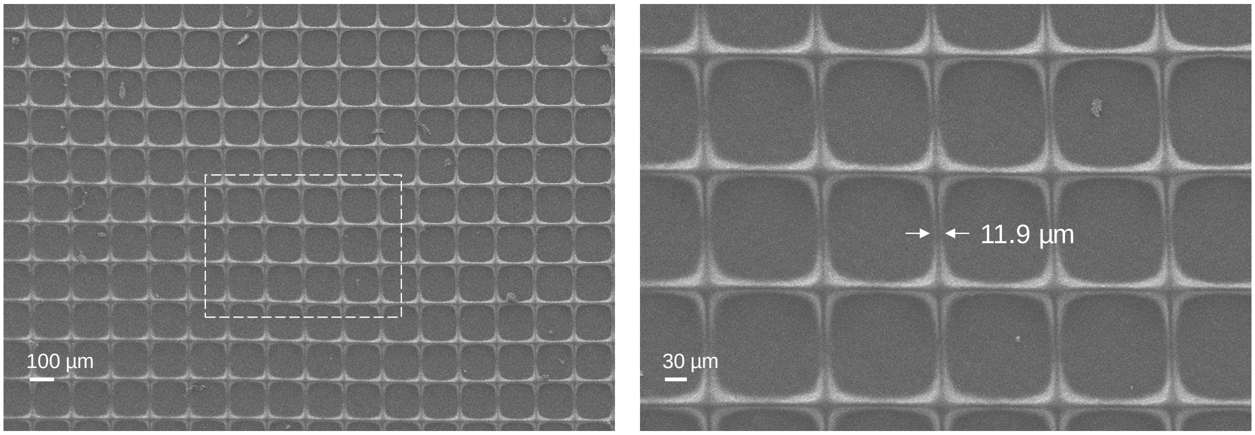

Fig. S3. Minimum feature size of microstructures printed by Lens 2. SEM of square kaleidoscopic interwoven patterns (***a***'/***p*** = 1) with the minimum feature size of 11.9 µm in Fig, S2B. These structures were produced with Lens 2, defined within the caption of Fig. 4 and in the Methods section).

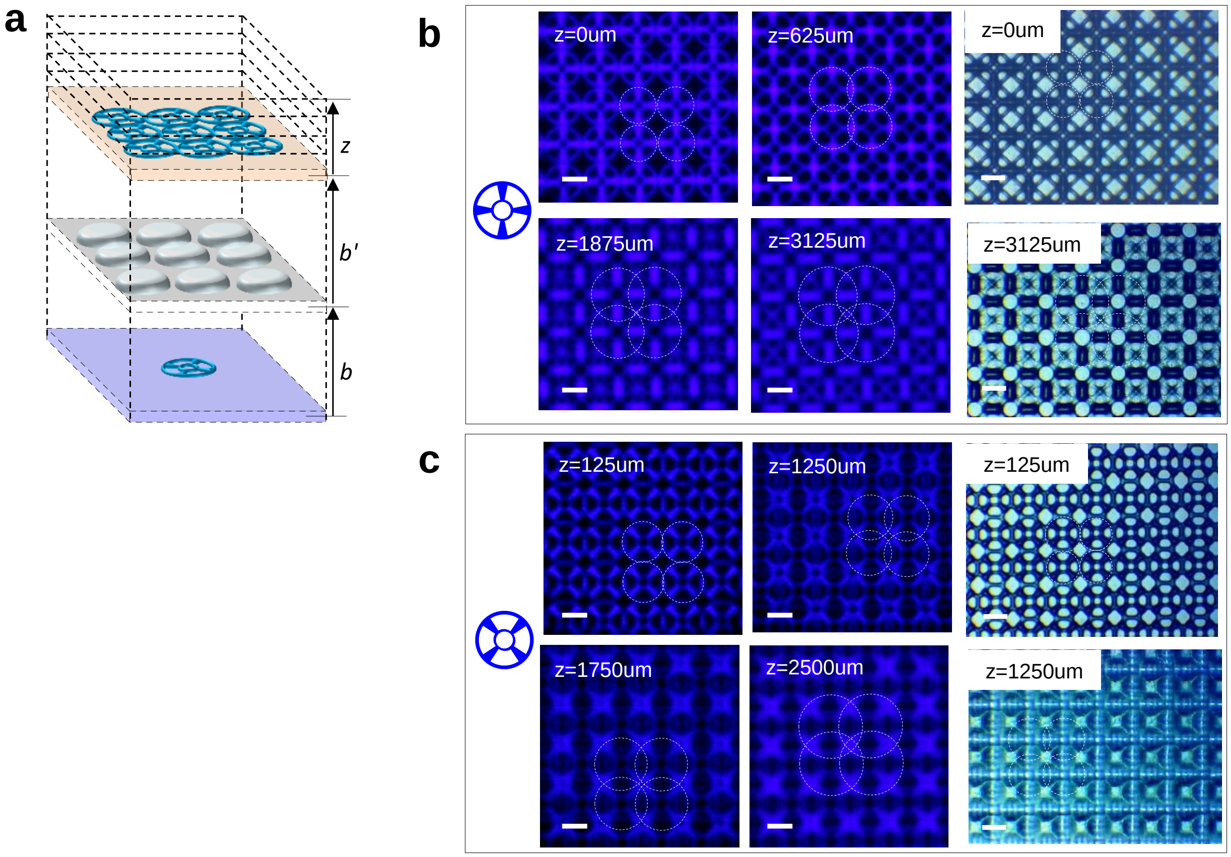

Fig. S4. Out-of-plane image-based kaleidoscopic patterns with periodic optical distribution at different propagation distance along *z* (*z* ≥ *b'*). **(A)** Replicated patterns with an array-lens, including distance definitions. **(B-C)** Various kaleidoscopic images and printed structures. Object image is provided on the left. All scale bars are 100 μm.

Dynamic image-based kaleidoscopic patterns

Unlike the illumination-based approach in prior work (69), the kaleidoscopic approach in this work stems from the superposition of multiple output images and enables more controllable and predictable patterns through combinations of the dynamic input image and lens array imaging. Figures S2-4 shows representative output patterns and printings created by modulating the geometry, and size of the input images, and propagation distance (***z***) via the lens array. Kaleidoscopic interwoven lattices were printed with Lens 2 with a minimum feature size of ~ 12 μm over an exposure area up to 100 mm^2 (Figs. S3). First, the kaleidoscopic images in Fig. S2-3 were produced by adjusting the projection image shapes and sizes from 0.92 to 3.66 mm with a ***D*** of 0.082. Here, the focal plane of the digital microscope camera coincided with the imaging plane of the lens array (***z*** = ***b'***). Second, the vertical set position of the digital microscope camera was changed until the lens array produced out-of-plane kaleidoscopic patterns. Then, the distance between the focal plane of the microscope and the imaging plane of lens array was gradually increased. The images in Fig. S4 were captured as the distance varied (***z*** ≥ ***b'***).

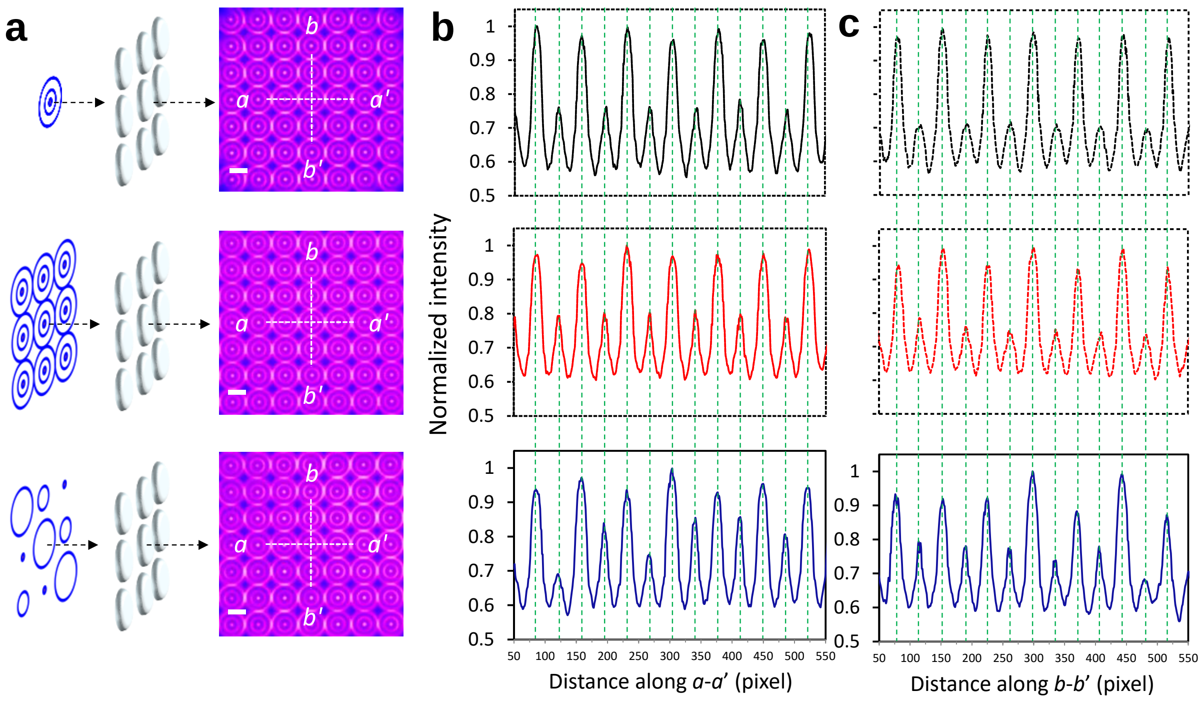

Fig. S5. Intensity profiles of integral imaging patterns with compressive multi-projection. (A) Different imaging configurations and optical microscope images. Plot of normalized gray value versus distance along **(B)** line a-a' and **(C)** line b-b' in (A). A close analysis of captured images and their corresponding intensity profiles reveals that the synthetic pattern with three-decomposed EIs generates slightly asymmetric profiles as compared to that of synthetic patterns with identical EIs. This is presumably due to the small discrepancy of reconstructed images generated by the overlap of spatially non-uniform images from slightly different perspectives, which may be related to the registration accuracy of EIs. All scale bars are 100 μm.

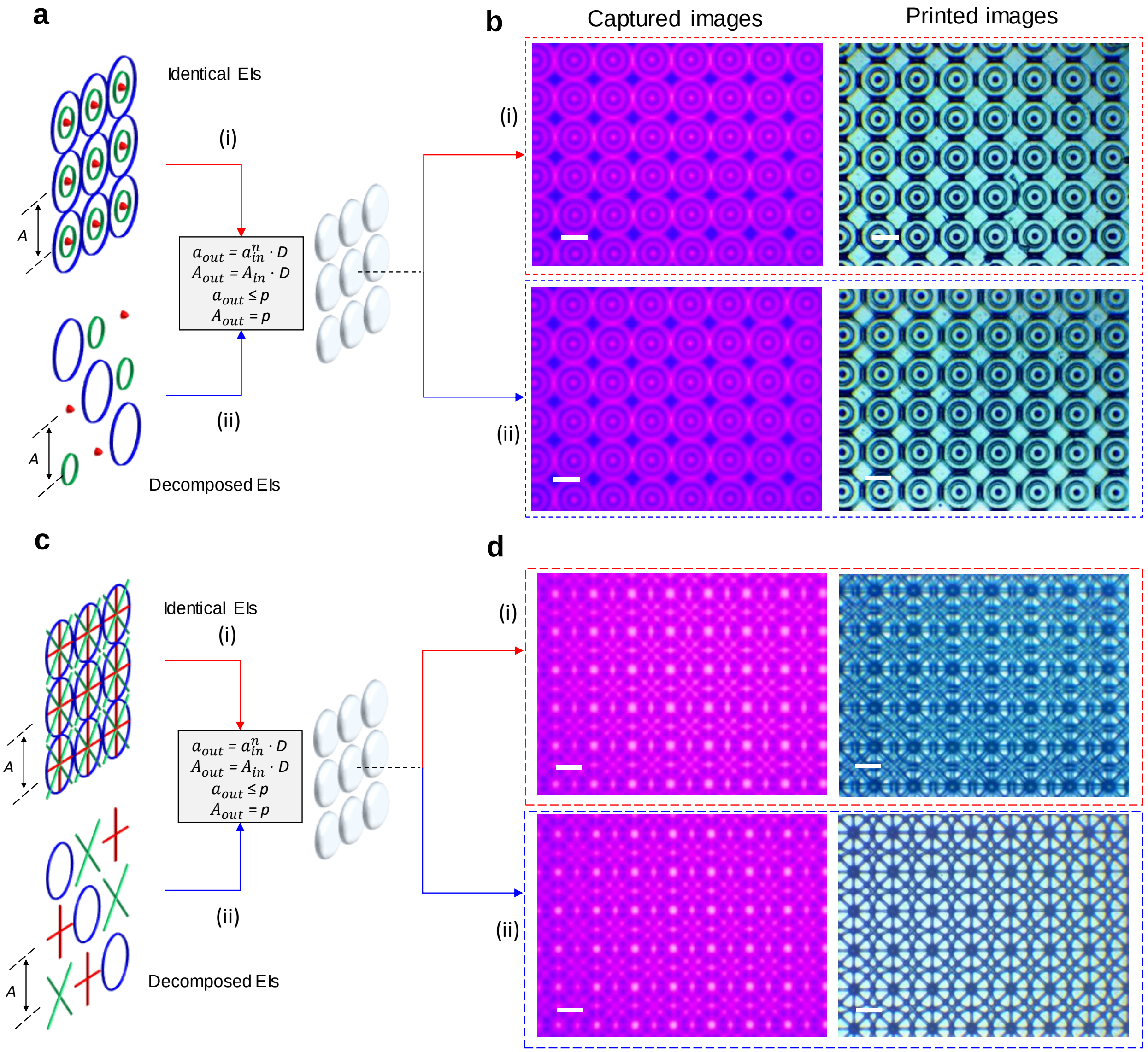

Fig. S6. Integral imaging mechanism. (A, C) Image replication and multiple EIs capture through the lens array. **(B, D)** Optical microscope-captured images and corresponding polymerized microstructures. All scale bars are 100 µm.

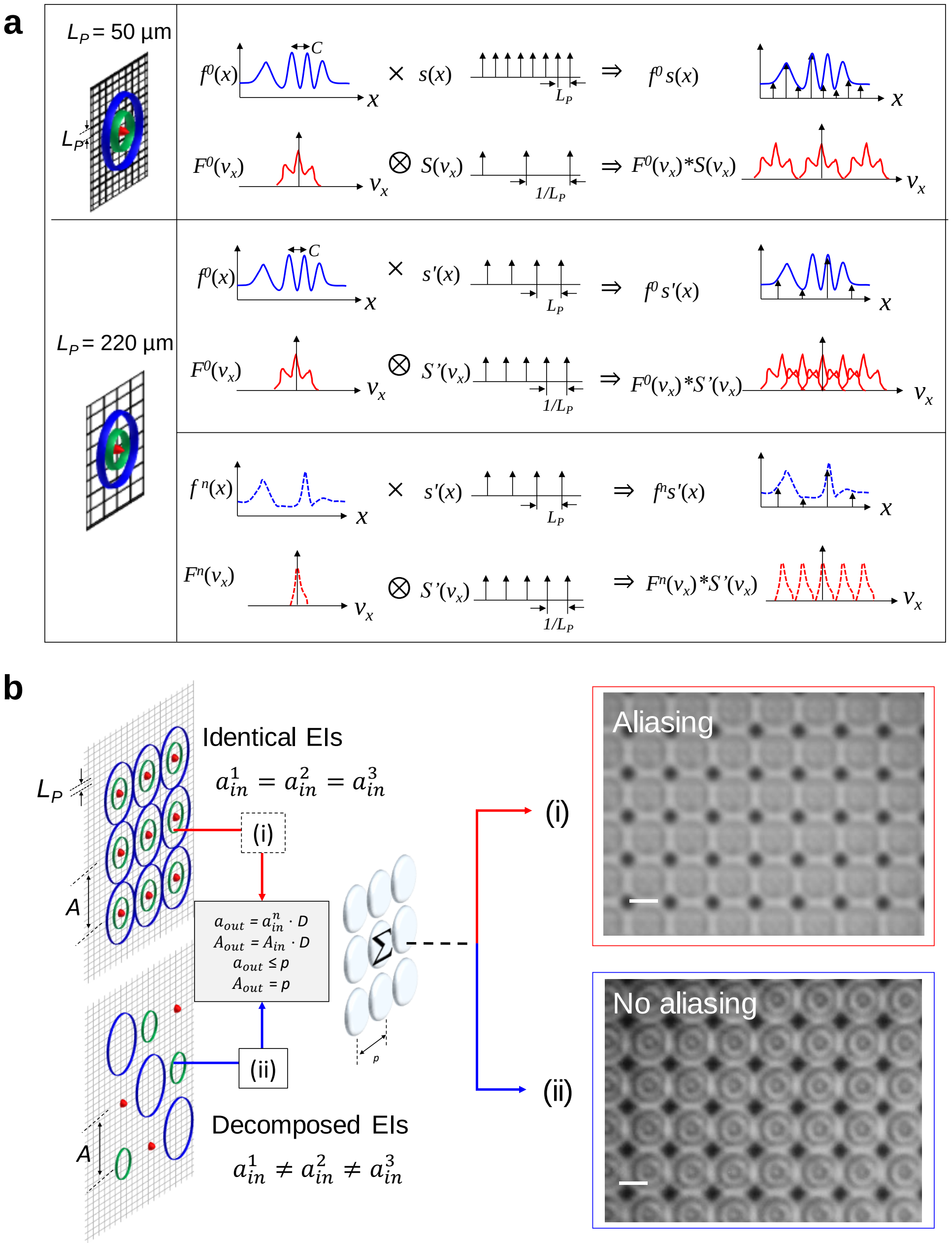

Fig. S7. Compressive integral imaging. (A) Frequency analysis of the image reconstruction for large (1280 × 800 with L_P of 50 μm) and low display bandwidth (1024 × 600 with L_P of 220 μm). Here, $f(x)$ and L_P represent the target object and the projected pixel size of constituent digital microdisplay, respectively. $s(x)$ and v_x denote the sampling function of comb(x/L_P) caused by the effect of the pixel sampling and the spatial frequency (= $1/C$) of the target image, respectively. **(B)** Output imaging patterns created by a microdisplay with an L_P of 220 μm. (i) Integral imaging with identical EIs of a concentric circular grating. (ii) Integral imaging with the three decomposed EIs for synthesis into the concentric circular grating on the imaging plane through the lens array. All scale bars are 100 μm.

Integral imaging patterns with compressive multi-projection

According to a simple one-dimensional model assumption in Fig. S7, the pixel values of the display image $\boldsymbol{a}_{\boldsymbol{in}}^{\boldsymbol{n}}(\boldsymbol{x})$ can be represented by $f^n(\boldsymbol{x})\mathrm{comb}(\boldsymbol{x}/\boldsymbol{L_P}) * \mathrm{rect}(\boldsymbol{x}/\boldsymbol{L_P})$, where $f^n(\boldsymbol{x})$ and $\boldsymbol{L_P}$ represent the target object and the projected pixel size of the constituent digital display, respectively. The Fourier transform of $\boldsymbol{a}_{\boldsymbol{in}}^{\boldsymbol{n}}(\boldsymbol{x})$ is represented by $\boldsymbol{A}_{\boldsymbol{in}}^{\boldsymbol{n}}(\boldsymbol{v_x}) = \left|\boldsymbol{L_P}^2\right| F^n(\boldsymbol{v_x}) * \mathrm{comb}(\boldsymbol{v_x}\boldsymbol{L_P})\mathrm{sinc}(\boldsymbol{v_x}\boldsymbol{L_P})$, where $\boldsymbol{v_x}$ is the spatial frequency (= 1/$\boldsymbol{C}$) of the target image. Considering the Nyquist sampling criteria ($\boldsymbol{v_N}$ = 1/2$\boldsymbol{L_P}$), a digital micromirror device (DMD)-based display unit ($\boldsymbol{L_P}$ of ~ 50 μm) can provide enough spatial resolution ($|\boldsymbol{v_x}| < \boldsymbol{v_N}$) to prevent the aliasing (i.e., spectral overlap) in integral imaging with both identical and decomposed EIs, thereby enables comparable reconstruction performance, as shown in Fig. 2C. However, a microdisplay with $\boldsymbol{L_P}$ of ~ 220 μm results in the aliasing from the integral imaging of identical EIs (Fig. S7B-i) due to insufficient spatial resolution ($|\boldsymbol{v_x}| > \boldsymbol{v_N}$). Herein, the compressive integral projection used to decompose the high-frequency spatial component of the initial target image (i.e., $|\boldsymbol{v_x}| < \boldsymbol{v_N}$) can provide a solution that can restore the desired target image. As shown in Figure S6B-ii, the integration of micro-optical elements into relatively cheap display devices can resolve high frequency components without aliasing effects, and is expected to facilitate low-cost mass-production. Here, we arranged EIs of 9 × 9 with distance $\boldsymbol{A}$ of 1.83 mm showing identical or decomposed images of the concentric circular grating. The input images in the case of Fig. S7B were generated via an LCD-based 3D printing system (Photocentric 3D Liquid Crystal LC10) with an $\boldsymbol{L_D}$ of ~ 220 μm, $\boldsymbol{M}$ of 1, and $\boldsymbol{A_S}$ of 2 × 10^4 mm^2. In the relationship $[\boldsymbol{a_{out}}] = [\boldsymbol{H}][\boldsymbol{a}_{\boldsymbol{in}}^{\boldsymbol{n}}]$ as depicted in Fig. 2B, the transfer matrix $\boldsymbol{H}$ was determined by its elements $\boldsymbol{h_{m,n}}$, which represented the impulse response function of the unit-lens in the lens array, where $\boldsymbol{m}$ and $\boldsymbol{n}$ represent the numbers of the unit-lens within the lens array in the horizontal and vertical directions, respectively. Considering the demagnification ($\boldsymbol{D}$) of the object image by the unit-lens, the $\boldsymbol{h_{m,n}}$ of the lens array can be defined as $\mathrm{rect}(\boldsymbol{xD}/\boldsymbol{p}) * \mathrm{comb}(\boldsymbol{x}/\boldsymbol{p})$, where rect($\boldsymbol{xD}/\boldsymbol{p}$) and comb($\boldsymbol{x}/\boldsymbol{p}$) are the window function of the unit-lens and the modulation of the entire lens array composed of many identical unit-lenses, respectively. In this study, all $\boldsymbol{h_{m,n}}$ were assumed to be identical. The transfer function can be described as $\mathrm{sinc}(\boldsymbol{v_x}\boldsymbol{p}/\boldsymbol{D})\mathrm{comb}(\boldsymbol{v_x}\boldsymbol{p})$ through a Fourier transform of the impulse response, where $\boldsymbol{v_x}$ is the spatial frequency of the displayed images. Thus, the lens array imaging can increase the amount of the transferable spatial information by a factor of $\boldsymbol{D}$.

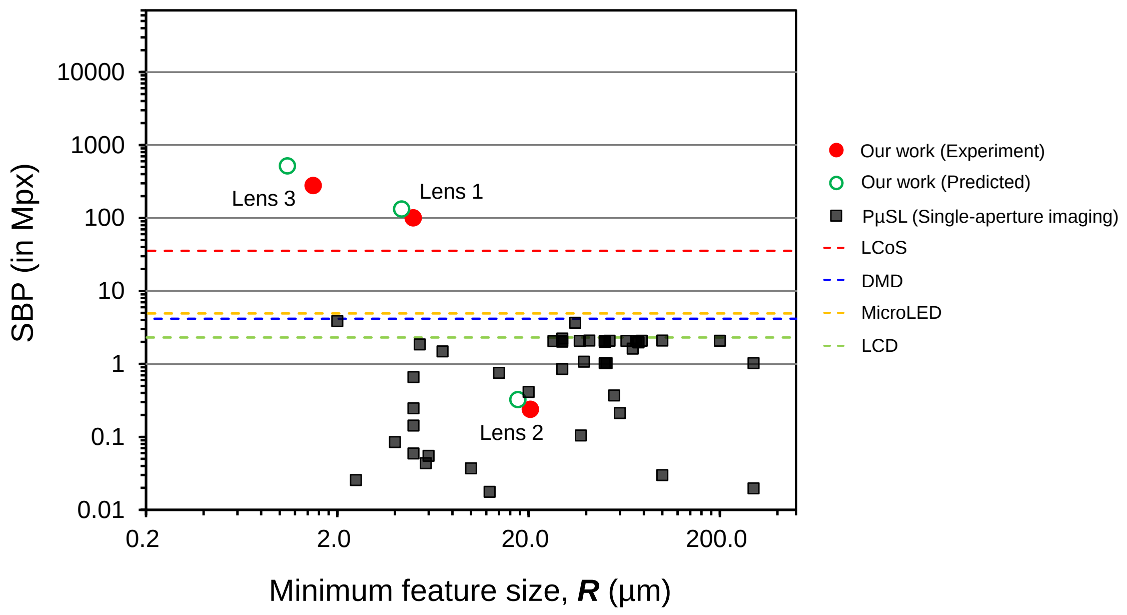

Fig. S8. SBP-resolution summary. Plotted data points show the specific published results of the current PµSL systems. The dashed line represents analytical scaling equations grouped by digital microdisplay devices of LCoS, DMD, MicroLED, or LCD. The red and green circles represent experimental results as well as the authors' calculations for ideal results considering the potential of integral lithographic system, respectively. Type or paste caption here. Create a page break and paste in the Table above the caption.

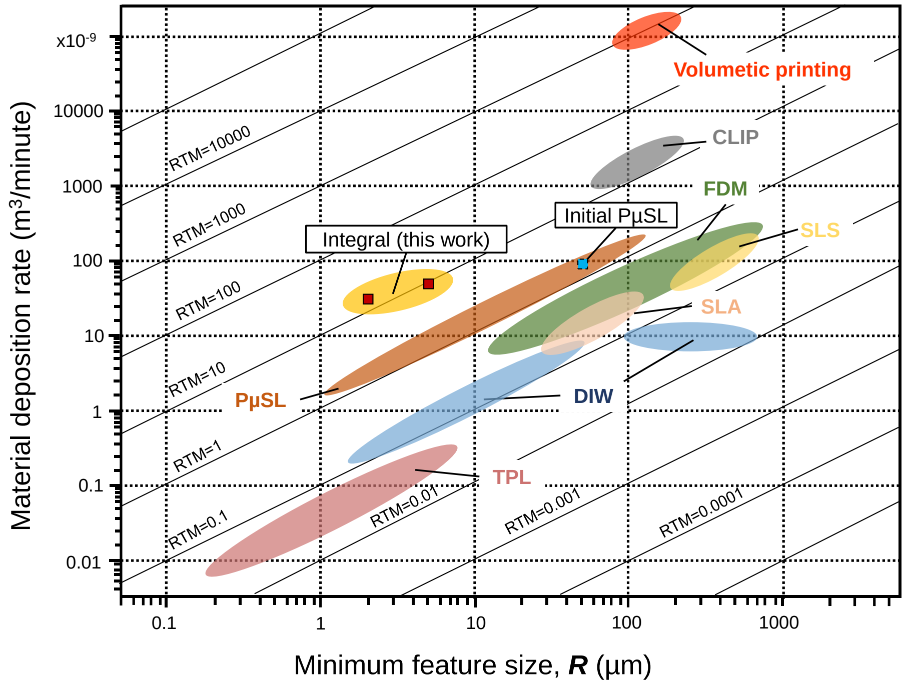

Fig. S9. Performance comparison of the integral lithographic system to existing polymer-based 3D printing methods.

An analysis of maximum material deposition rate (m^3/min) and minimum feature size (µm) is an additional figure of merit (FOM) to compare the system performance of integral lithography to existing polymer-based 3D printing methods. Based on our previous analysis (70), the resolution-to-manufacturing time ratio (RTM, expressed with units of $10^{-3}m^2$/min) was defined as a function of the spatial resolution and time for manufacturing to quantitative compare the efficiency of different 3D printing techniques.

$$\text{RTM} = \frac{\text{Spatial resolution}}{\text{Time for manufacturing}} \cong \boldsymbol{r} \cdot \boldsymbol{P} = \frac{1}{\boldsymbol{R}} \cdot \frac{\boldsymbol{V}}{\boldsymbol{t}} \qquad \text{(S1)}$$

Here, $\boldsymbol{r}$ is the best spatial resolution achieved within the printing technology, expressed as the inverse of the minimum feature size $\boldsymbol{R}$. The delivery rate $\boldsymbol{P}$ of the material being printed is a function of the volume $\boldsymbol{V}$ delivered per unit of time $\boldsymbol{t}$. In additive manufacturing field, the order of magnitude of $\boldsymbol{R}$ and $\boldsymbol{P}$ are µm and mm^3/minute respectively. Hence, the RTM ratio is expressed in $10^{-3}m^2$/minute for an easier comparison between different technologies. Note that $\boldsymbol{R}$ or $\boldsymbol{P}$ may vary depending on the materials delivered, the geometry, and the printing parameters.

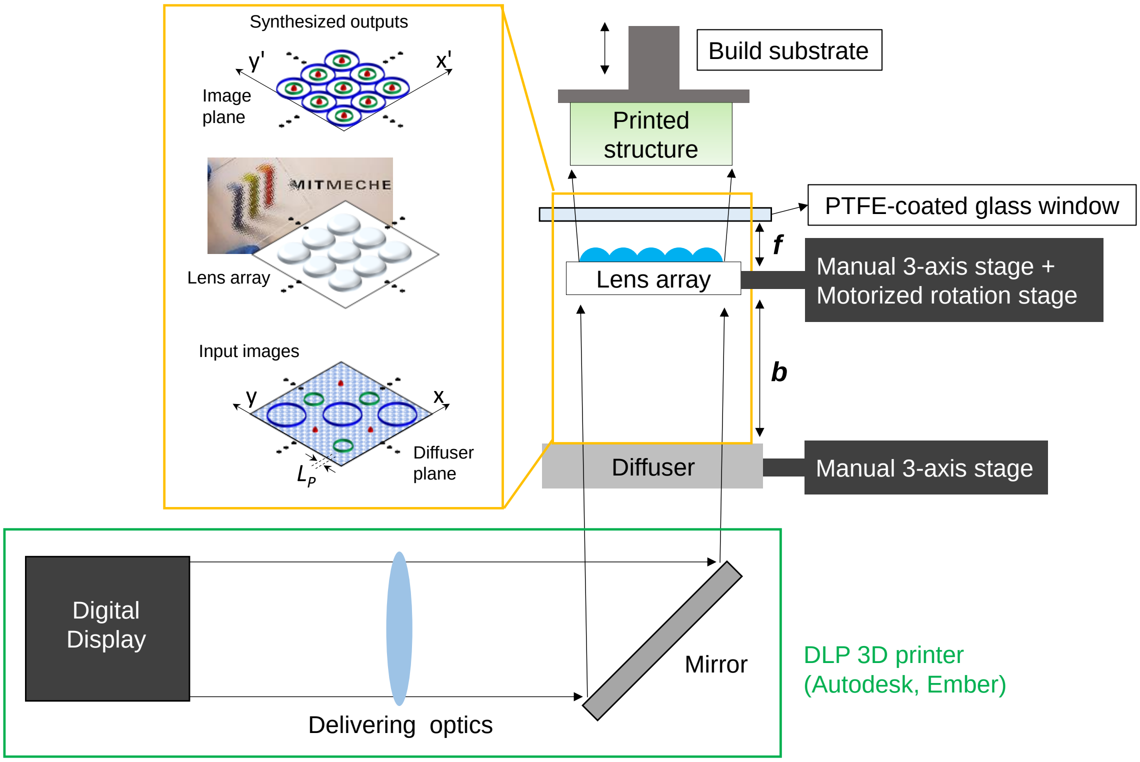

Fig. S10. Schematic diagram of the integral lithographic system.

The lens array was mounted on a motorized rotation stage (Thorlabs, PRM1Z8) combined with a manual 3-axis translation stage with 25 µm translation resolution, which allows for fine longitudinal adjustment along the z-axis and control of the angular offsets. The diffuser was also mounted on a manual 3-axis translation stage with 25 µm translation resolution. For Lenses 1 and 2, we used a 1 mm-thick glass plate, covered with optically-clear PTFE FEP with a thickness of 50 µm (CS Hyde, Lake Villa, IL, USA) for a separation layer of the printed layers. For Lens 3, particularly, the thick glass plate was replaced with a thin glass plate with a thickness of 80 – 115 µm (Thorlabs, CG00K1-Cover Glasses, #0 Thickness) considering the short focal length.

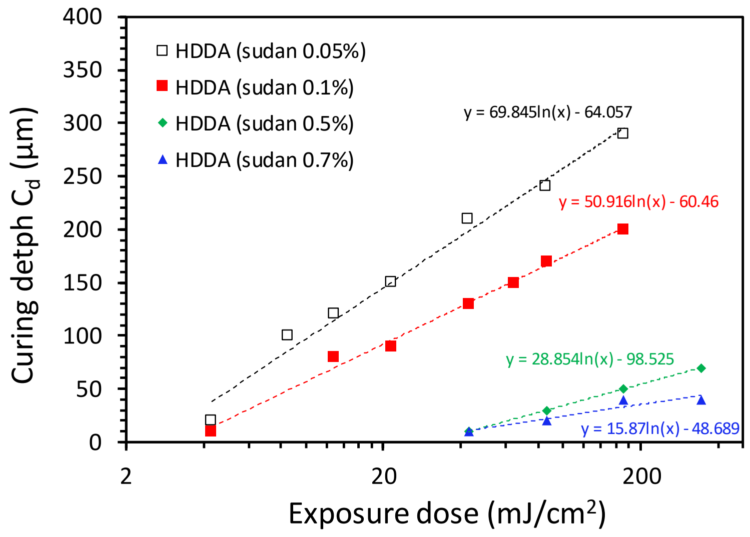

Fig. S11. Cure depth versus energy for UV absorber (Sudan 1) containing 0.05 - 0.7% (w/w) in hexanedioldiacrylate (HDDA).

The spatial resolution and photopolymerization depth study

Because the current system is based on the projection microstereolithography (PµSL) technique, in-plane resolution (lateral direction) is limited by the performance of the projection system (e.g., an optical wavelength of the illuminating light, the pixel size of a digital microdisplay, and the effective ***NA*** of a lens) and the photopolymerization kinetics (9, 71). The details about the in-plane resolution in the current system are described in the main text. On the other hand, vertical resolution can be determined by two main parameters such as the resolution of the vertical translational stage and the photopolymerization depth in vertical direction. The current system can faithfully achieve a vertical translational stage resolution of 5 µm. Also, the PµSL techniques utilize photochemical effect to convert liquid monomer into solid polymer in which a photopolymerization process involves chemical factors such as concentration of photoinitiator and photoabsorber, diffusivity of photoinitiator, and quantum yield. When UV radiation enters the photopolymer resin, it is absorbed by the resin. The absorbed energy distribution inside the resin surface can be determined by means of the Lambert-Beer absorption law. The vertical resolution is determined by light penetration depth ($\boldsymbol{D_p}$) (defined as the depth within the resin where irradiation drops to 1/e of the intensity on the surface) and the photopolymerization depth ($\boldsymbol{C_d}$). The detailed theoretical model for the photopolymerization process has been described in Refs 5-6. Here, we investigated the experimental study for $\boldsymbol{C_d}$ to obtain the process condition for the printing of the desired structure. The experimental result of the photopolymerization depth study provides empirical working curves to be used to determine the appropriate light intensity and exposure time in the current system. The relationship between the cure depth $\boldsymbol{C_d}$ and the applied exposure dose $\boldsymbol{E}$ is expressed by

$$\boldsymbol{C_d} = \boldsymbol{D_p} \ln\left(\frac{\boldsymbol{E}}{\boldsymbol{E_c}}\right), \quad \text{(S2)}$$

where $\boldsymbol{E_c}$ is the critical exposure dose. Equation S2 defines the working curve that is used to control the polymerization thickness. Note that the horizontal axis is logarithmic scale of exposure and exposure is obtained by multiplying light intensity and exposure time ($\boldsymbol{E} = \boldsymbol{I} \times \boldsymbol{t}$).

Figure S11 has measurements for the cure depth $\boldsymbol{C_d}$ as a function of exposure dose $\boldsymbol{E}$, plotted according to Equation S2 so that the $\boldsymbol{D_p}$ parameter can be measured from the slope and the $\boldsymbol{E_c}$ parameter can be obtained from the energy dose intercept. These measurements were taken for UV absorber (Sudan 1) containing 0.05 - 0.7% (w/w) in hexanedioldiacrylate (HDDA).

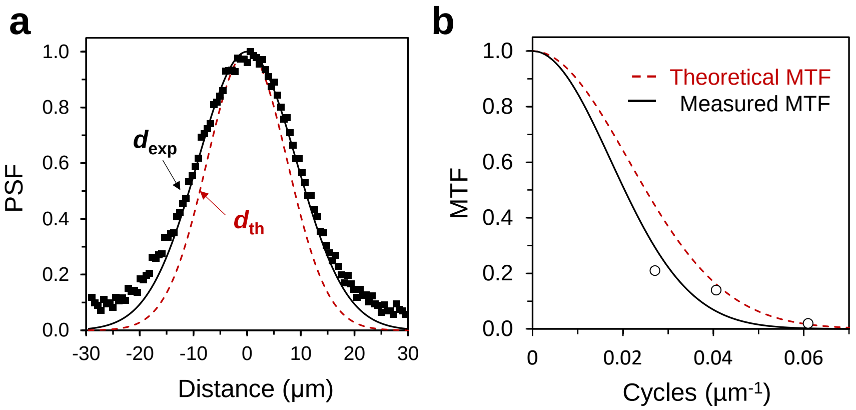

Fig. S12. Characterization of the imaging quality in the integral lithographic system. (**a**) Point spread function (PSF) estimate of unit lens of Lens 2. The theoretical calculated and experimental determined PSF are shown as a red dashed line and black dots (black solid line fit to Gaussian distribution), respectively. (**b**) Modulation transfer function (MTF) for the same lens array. The theoretical calculated and experimental determined MTF are shown in red dashed and black solid line, respectively.

To estimate the imaging quality of the integral lithographic system, we determined and interpreted the PSF and MTF through Lens 2 as a quantitative measure of the resolution and line pattern contrast limits. In this work, we exposed one-pixel illumination (~ 50 µm) of the current DLP projector as an image of the point source to individual unit lenses. We then captured the unit lens' PSF through high index media (PTFE-coated glass window) using a custom-built microscope composed of a 4× objective and digital CMOS sensor (AmScope MU500). The theoretical PSF of a lens array' unit with $\boldsymbol{NA}$ = 0.014 for 405 nm source is computed to a feature size of $\boldsymbol{d_{th}}$ = 17.6 µm with Rayleigh criterion ($1.22\lambda/2\boldsymbol{NA}$) and the full width at half maximum (FWHM) of experimental PSF was measured to $\boldsymbol{d_{exp}}$ = 21.5 µm with a Gaussian approximation (Fig. S12a). Since the Airy pattern falls rather slowly to zero with increasing distance from the center and the resultant spot size was undefined, we used a Gaussian approximation as an alternative measure of the spot size by ignoring the relatively small outer rings of the Airy pattern. We obtained the theoretical and measured MTF by Fourier transforming the theoretical and measured PSF, respectively (Fig. S12b). Here, the discrepancy between the theoretical and measured PSF is likely to be caused by the residual blur from the custom-built microscope optics and the aberrations of the lens array.

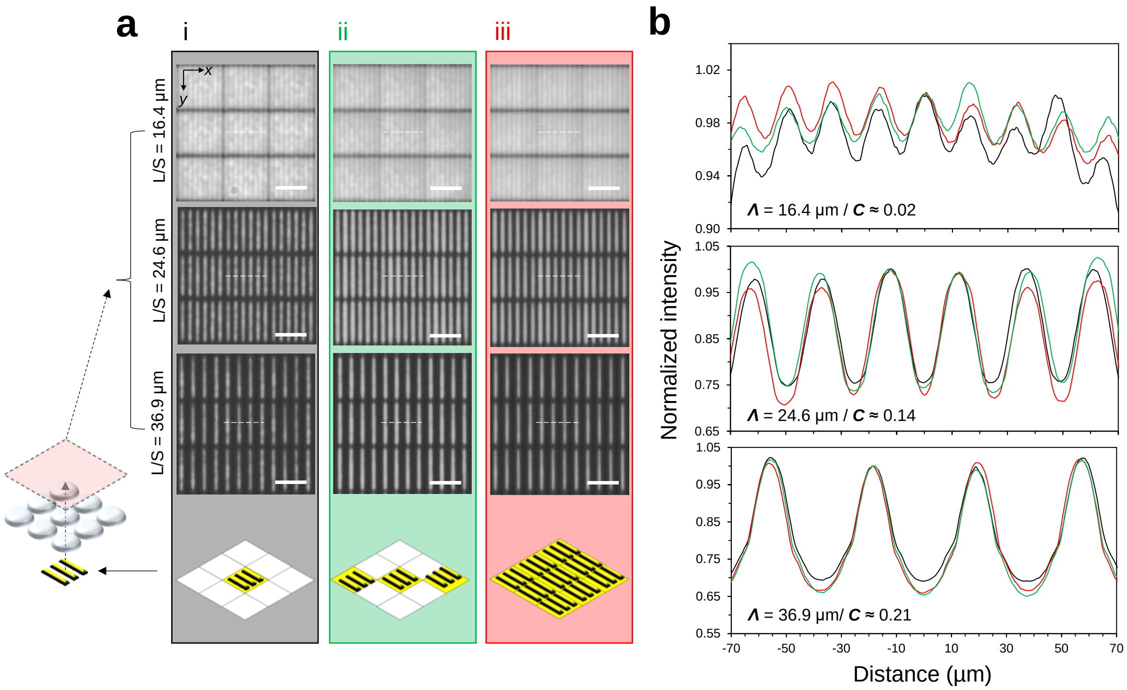

Fig. S13. Image quality evaluation at the distal boundary of the extended field of view. (**a**) Images taken with the different integral imaging patterns; parallel replication of a single object image (i) and integral imaging patterns with three decomposed EIs (ii) and identical EIs (iii). These captured images were quantitatively analyzed using the imaging software ImageJ. (**b**) Lateral line intensity profiles with a different grating period Λ. Intensity profiles were measured to check the image quality of integral imaging patterns at the edge boundary of each lens array. All scale bars are 100 μm.

The MTF at given spatial frequencies can be experimentally measured as the contrast $\boldsymbol{C}$ (defined as $(\boldsymbol{I}_{max} - \boldsymbol{I}_{min})/(\boldsymbol{I}_{max} + \boldsymbol{I}_{min})$) of the sinusoidal grating of the specified spatial frequency through the optical system, where $\boldsymbol{I}_{max}$ and $\boldsymbol{I}_{min}$ the maximal and minimal intensity values of the image of the line grating, respectively. We also measured the MTF of the integral lithographic system by projecting line gratings of three different spatial frequencies on Lens 2 in different imaging modes such as parallel replication of a single object image and integral imaging patterns with three decomposed and identical elemental images (Fig. S13). We confirmed that the measured $\boldsymbol{C}$ (circle dots in Fig. S12b) was comparatively consistent with the theoretical and measured MTF curve (Fig. S12b).

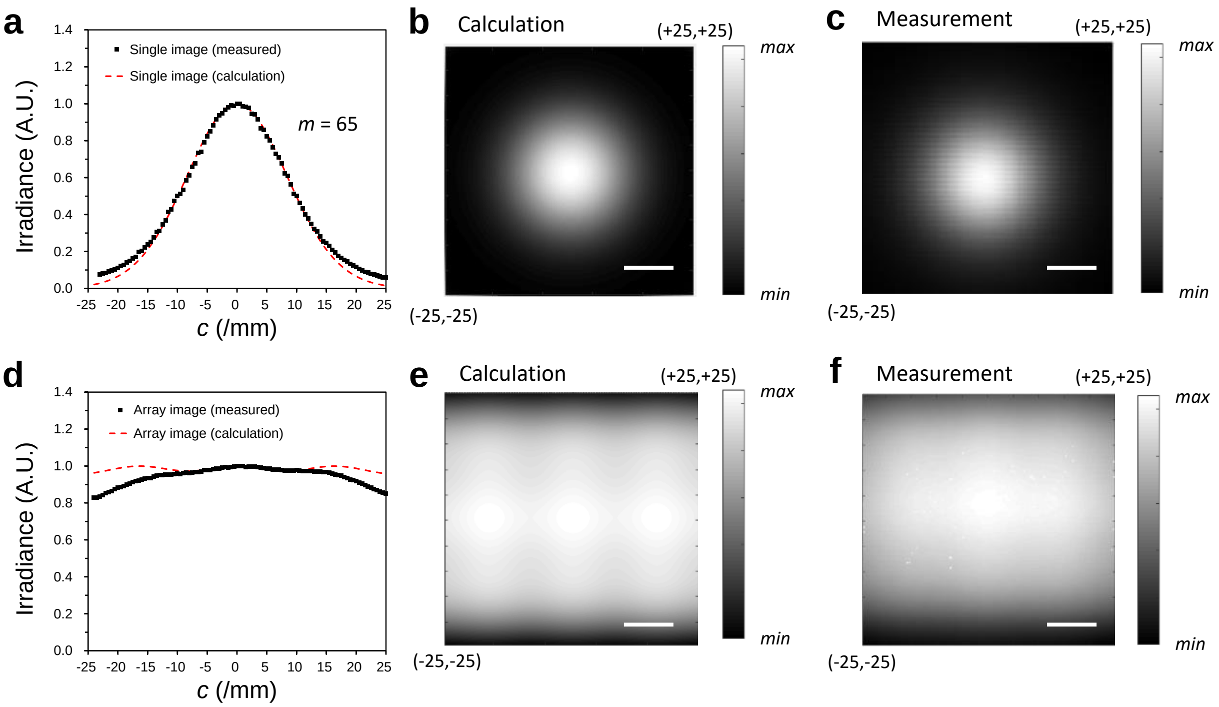

Fig. S14. The calculated and measured illumination distributions for the current integral imaging system. (A) The irradiance distribution (normalized to its maximum value) along the horizontal direction $\boldsymbol{C_x}$ direction at the center of vertical direction $\boldsymbol{C_y}$ for $\boldsymbol{a}$ = 9 mm and $\boldsymbol{b}$ = 68.75 mm. The dotted and dashed curves show the measured and calculated irradiance patterns of a single image source, respectively. **(B-C)** Calculated and measured 2D irradiance distributions for a single image source, respectively. **(D)** The corresponding irradiance distribution for a square array of 5 × 3 circular sources with $\boldsymbol{a}$ = 9 mm and $\boldsymbol{A}$ = 10 mm. **(E-F)** Calculated and measured 2D irradiance distributions for a rectangular array of image sources, respectively. We note that the irradiance was measured at the imaging distance $\boldsymbol{b}$ = 68.75 mm without the array-lens. All scale bars are 10 mm.

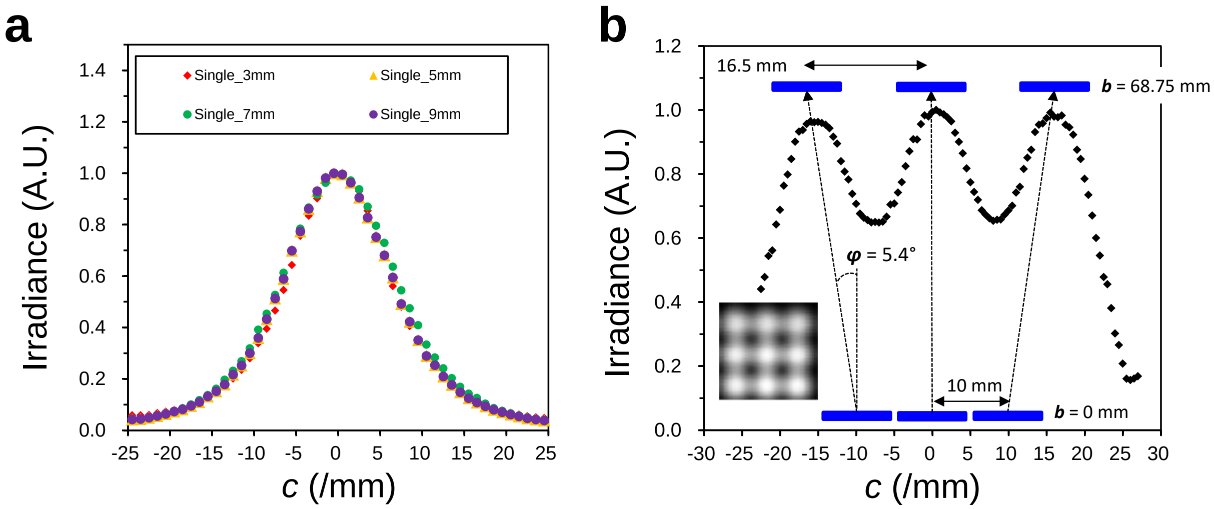

Fig. S15. The irradiance distribution. (A) The normalized illumination distribution according to the size variation of ***a***. **(B)** The measured divergence angle ***φ*** in our system. The irradiance distribution (normalized to its maximum value) was measured at the imaging distance ***b*** = 68.75 mm without the diffuser and the lens array.

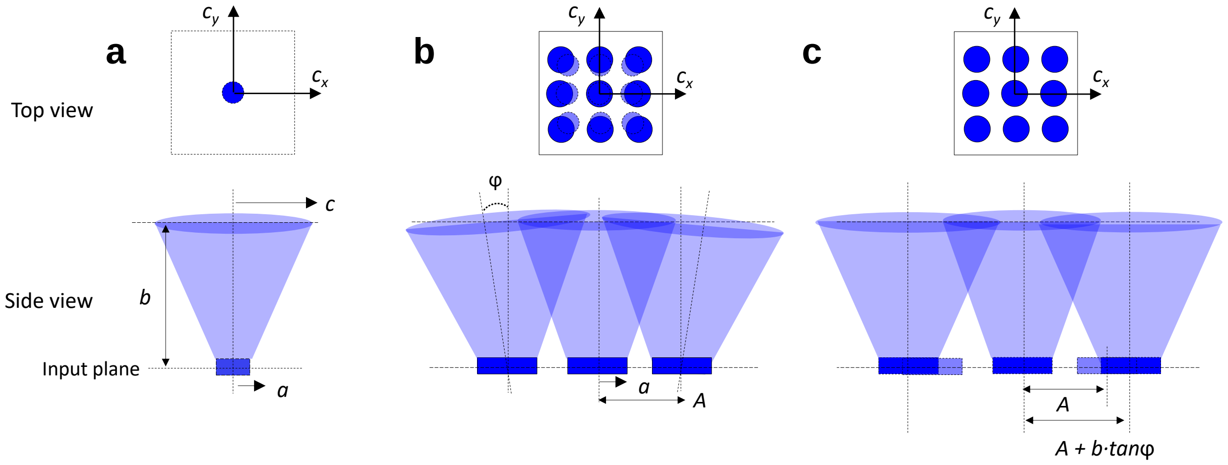

Fig. S16. Conceptual schematics for analyzing the illumination distribution between a single image source and array image sources. (A) Illumination distribution of the single image source. **(B)** Actual illumination distribution and propagation of array image sources in our system. **(C)** The paraxial approximation for simplicity in this study.

Illumination distribution

To describe a simple analytical equation for the illumination distribution in our system, we assume that the input image produces an incoherent illuminating source and a nonperfect Lambertian distribution defined as a cosine-power function of $P(r,\theta) \propto P_0(r)cos^m\theta$ where $\boldsymbol{\theta}$ is the radiating angle, $\boldsymbol{m}$ is the directionality, $\boldsymbol{P}_0(\boldsymbol{r}) = \boldsymbol{P}_0/\boldsymbol{r}^2$ is the radiant power on the axis at distance $\boldsymbol{r}$ from the image source when $\boldsymbol{\theta} = 0$ °, and $\boldsymbol{P}_0$ is proportional to $\boldsymbol{a}^2$. In Figs. S14A-C, the radiant power distribution projected at lateral position ($\boldsymbol{c_x}$, $\boldsymbol{c_y}$) over the illuminating plane at imaging distance $\boldsymbol{b}$ from a single image source is described by radiometric theory with Cartesian coordinates (72-74). Accordingly, the practical approximation for the radiant power distribution of a single image source right below the array-lens can be described as

$$P(a,b,c) \propto \frac{a^2b^m}{\left[\left(b^2+c_x^2+c_y^2\right)\right]^{\frac{m+2}{2}}}. \tag{S3}$$

We measured the illumination distribution over the $\boldsymbol{c_x}$-$\boldsymbol{c_y}$ plane with variations of size $\boldsymbol{a}$ when $\boldsymbol{b}$ = 68.75 mm, and further fitted the measured irradiance distribution to determine the constant $\boldsymbol{m}$. Based on the measurements of the radiant power distribution for a single image source along the $\boldsymbol{c_x}$ axis at $\boldsymbol{c_y} = 0$ for a selected value of $\boldsymbol{a}$ = 9 mm, we extracted the fitting constant $\boldsymbol{m}$ = 65 in our system (Figs. S14A and 15A). Also, we noticed that the constant $\boldsymbol{m}$ significantly depends on the radiating characteristic of the diffusing optics and the divergence of the delivering optics, rendering it effectively independent of the size $\boldsymbol{a}$ (Fig. S15A). Consequently, we assume that all input images have equal values of $\boldsymbol{m}$ in this study. From the illumination distribution analysis with a single object source, we derive the approximated equation of irradiance superposition to estimate the illumination distribution from an array object source. After considering the measured divergence angle $\boldsymbol{\varphi}$ caused by the delivery optics of the initial printing system used in this study (Fig. S15B), the illumination distribution from the array object source is approximated

by the summation of the irradiances of a matrix of $\boldsymbol{N} \times \boldsymbol{M}$ (where $\boldsymbol{N}$ and $\boldsymbol{M}$ are odd numbers) images,

$$P(a,b,c) \propto a^2 b^m \cdot \sum_{j=1}^{M} \sum_{i=1}^{N} \left\{ b^2 + \left[c_x - (N+1-2i)\left(\frac{A+b\cdot tan\varphi}{2}\right)\right]^2 + \left[c_y - (M+1-2i)\left(\frac{A+b\cdot tan\varphi}{2}\right)\right]^2 \right\}^{-\frac{m+2}{2}}. \quad \text{(S4)}$$

For the simple analytic approach, we used a paraxial approximation that the divergence angle shifts only the distance between array image sources and thereby model the illumination propagation to the array-lens without divergence (Fig. S16). In a simple circle array object source with $\boldsymbol{N} \times \boldsymbol{M} = 5 \times 3$, the calculated and measured illumination distributions for a rectangular arrangement of image sources is shown in Figs. S14E and F when $\boldsymbol{a}$ = 9 mm, $\boldsymbol{A}$ = 10 mm, and $\boldsymbol{\varphi}$ = 5.4 °. We believe that the discrepancy of the calculation and measurement at the edges may originate from the small divergence angle $\boldsymbol{\varphi}$ caused by the delivering optics in our initial printing system.

Reference	Type of digital micridisplay	Display area (mm^2)	Optics	Minimum feature size (μm)	Maximum Projection Area/exposure (mm^2)	Printing Methods
2,12,14	LCoS 1920 × 1080<br>pixel: 8 um	15.36 x 8.64	6:1 reduction lens	10	2.56 x 1.44	top-down
9	DMD 1280 x 1024<br>pixel: 14–17 um	17.9 x 14.3<br>21.76 x 17.4	5:1 reduction lens	3.4	3.58 x 2.86<br>4.3 x 3.5	top-down
11, 87,<br>Carbon M1	DLP	10.3 x 7.7	N/A	M1: 75<br>Experiment: 50	141 x 79	Continuous bottom-up
13	DMD 1920 × 1080<br>pixel: 5 um	9.6 × 5.4	1:4 projection lens	200	384 x 216	
17	LCoS<br>Pixels: 0.3 x 10^4	N/A	Holographic imaging system	100	10 x 10	Volumetric printing
18	DLP<br>Pixels: 1280 x 800	N/A	Axial imaging system	300	up to 55 mm	Volumetric printing
57	DMD 1024 x 768<br>pixel: 13.68 um	14 x 10.5	5:1 reduction lens	2.5	2.8 x 2.1	Maskless Projection Lithography,<br>protein adsorption by photobleaching
75	DMD 1024 x 768<br>pixel: 13.68 um	14 x 10.5	N/A	50	N/A	top-down
76	LCD<br>pixel: 26 x 24 um^2	N/A	N/A	20	15 x 11	top-down
77	DMD 1024 x 768<br>pixel: 13.68 um	14 x 10.5	NA 0.3<br>NA 0.13	10~30	1.95 × 1.95	top-down
78	DMD 1024 x 768<br>pixel: 13.68 um	14 x 10.5	10:1 reduction lens	5	1.4 x 1.05	top-down
50	DMD 1024 x 768<br>pixel: 13.68 um	14 x 10.5	1:1 lens	25~50	14.6 × 10.9	bottom-up
79	DMD 1024 x 768	N/A	Built in lens of<br>projector (CASIO XJ-S36)	300	48 x 36	multi-material<br>bottom-up
80	DMD 1920 x 1080<br>pixel: 10.8 um	N/A	2:1 reduction lens	5.4	6 x 8	top-down
81-83	DMD 1920 × 1080	N/A	N/A	5	4.6 x 3.5	top-down
84	DMD 1024 × 768<br>pixel: 13.6 um	14 x 10.5	1:1 projection lens	14	14 x 10.5	Maskless Projection Lithography
85	DMD 1024 x 768<br>pixel: 10.8 um	11 x 8.3	5:1 reduction lens	5	2.52 × 1.41	top-down
86	LCoS 1920 × 1080		7:1 reduction lens	10~15	1.2 × 2.2	top-down
51	DMD 1024x768<br>pixel: 13.6 um	14 x 10.5	10:1 reduction lens	20	1.4 x 1.05	Projection Printing
88	Asiga Pico Plus 27<br>DMD 912 x 1140<br>pixel: 7.6 um	0.45"	N/A	60	35 × 21.8	bottom-up
15	DMD 608 x 684<br>pixel: 7.6 um	0.3"	1:1 projection lens	100	4x2	top-down
89	DMD<br>pixel: 13.65 um	N/A	7:1 reduction lens	6	1.1 x 1.8	top-down
90	DMD	N/A	NA 0.30	2.5	0.4 x 0.4	Projection Printing
10	Epson PowerLite S5<br>LCD 800 x 600<br>pixel: 17 um	13.6 x 10	NA 0.30	3~5	1.36 x 1	bottom-up
91, 92	LCoS 1400 x 1050<br>pixel: 10.65 um	N/A	1.5:1 reduction lens	7.1	10 x 7.5	top-down
93	Optoma HD20 HD<br>DLP 4500	N/A	N/A	Optoma HD20 HD: 29.5<br>DLP4500: 51.2	Optoma HD20 HD: 56.7 × 35.4<br>DLP4500: 65.6 × 41	bottom-up
94	DLP 1024 × 768<br>pixel: 10.8 um	N/A	1:3 projection lens	30	32 × 24	
95	DLP 1024 x 768<br>pixel: 13.7 um	14 x 10.5	10:1 reduction lens	Theo. 1.37<br>Exp. 5.8	1.4 x 1.05	Grayscale gel lithography
96,<br>miicraft	DLP	N/A	N/A	Miicraft+ 56<br>MiiCraft 50: 30<br>MiiCraft 80: 41.5<br>MiiCraft 100: 53<br>MiiCraft 125: 65<br>MiiCraft 150: 78	Miicraft+: 43 × 27<br>MiiCraft 50: 57 x 32<br>MiiCraft 80: 80 x 45<br>MiiCraft 100: 102 x 57.5<br>MiiCraft 125: 125 x 70<br>MiiCraft 150: 150 x 84.4	bottom-up
Asiga Freeform<br>Pico 2	DLP	N/A	N/A	PICO 2-39: 39<br>PICO 2-50: 50<br>PICO 2 HD27: 27<br>PICO 2 HD37: 37	PICO 2-39: 51x32<br>PICO 2-50: 64x40<br>PICO 2 HD27: 51.8x29<br>PICO 2 HD37: 71x40	bottom-up
Autodesk<br>Ember	DMD 912 x 1140<br>pixel: 7.6 um	0.45"	1:7 projecgtion lens	50	64 x 40	bottom-up
DB9 Creator	DLP	N/A	N/A	DB9 creator: 70<br>B9 core 530: 30<br>B9 core 550: 50	DB9 creator: 102 × 78<br>B9 core 530: 57.6 x 32.4<br>B9 core 550: 96 x 54	bottom-up
Envision<br>Perfactory	DLP 1920 x 1080	N/A	N/A	vida: 73<br>vida HD: 50<br>Vida HD Crown & Bridge: 35	vida: 140 x 79<br>vida HD: 96 x 54<br>Vida HD Crown & Bridge: 90 x 50	bottom-up
Kudo3D<br>Titan	DLP 1920×1080	20	N/A	100<br>75<br>50	190 × 110<br>142 x 78.7<br>94 x 53.3	bottom-up

Table S1. Exposure area and minimum feature size of reported projection-based SLA printers. Commercial system performance is based on the manufacturer's specifications.

REFERENCES AND NOTES

1. T. A. Schaedler, A. J. Jacobsen, A. Torrents, A. E. Sorensen, J. Lian, J. R. Greer, L. Valdevit, W. B. Carter, Ultralight metallic microlattices. *Science* **334**, 962–965 (2011).

2. X. Zheng, H. Lee, T. H. Weisgraber, M. Shusteff, J. DeOtte, E. B. Duoss, J. D. Kuntz, M. M. Biener, Q. Ge, J. A. Jackson, S. O. Kucheyev, N. X. Fang, C. M. Spadaccini, Ultralight, ultrastiff mechanical metamaterials. *Science* **344**, 1373–1377 (2014).

3. K. Yu, N. X. Fang, G. Huang, Q. Wang, Magnetoactive acoustic metamaterials. *Adv. Mater.* **30**, 1706348 (2018).

4. D. Espinosa-Hoyos, A. Jagielska, K. A. Homan, H. Du, T. Busbee, D. G. Anderson, N. X. Fang, J. A. Lewis, K. J. Van Vliet. Engineered 3D-printed artificial axons. *Sci. Rep.* **8**, 478 (2018).

5. X. Zhou, C. Liu, Three-dimensional printing for catalytic applications: Current status and perspectives. *Adv. Funct. Mater.* **27**, 1701134 (2017).

6. C. M. Soukoulis, M. Wegener, Past achievements and future challenges in the development of three-dimensional photonic metamaterials. *Nat. Photon* **5**, 523–530 (2011).

7. R. L. Truby, A. Jennifer, J. A. Lewis, Printing soft matter in three dimensions. *Nature* **540**, 371–378 (2016).

8. Y. Zhang, F. Zhang, Z. Yan, Q. Ma, X. Li, Y. Huang, J. A. Rogers, Printing, folding and assembly methods for forming 3D mesostructures in advanced materials. *Nat. Rev. Mater.* **2**, 17019 (2017).

9. C. Sun, N. Fang, D. M. Wu, X. Zhang, Projection micro-stereolithography using digital micro-mirror dynamic mask. *Sens. Actuators A* **121**, 113–120 (2005).

10. R. Raman, B. Bhaduri, M. Mir, A. Shkumatov, M. K. Lee, G. Popescu, H. Kong, R. Bashir, High-resolution projection microstereolithography for patterning of neovasculature. *Adv. Healthc. Mater.* **5**, 610–619 (2016).

11. J. R. Tumbleston, D. Shirvanyants, N. Ermoshkin, R. Janusziewicz, A. R. Johnson, D. Kelly, K. Chen, R. Pinschmidt, J. P. Rolland, A. Ermoshkin, E. T. Samulski, J. M. DeSimone, Continuous liquid interface production of 3D objects. *Science* **347**, 1349–1352 (2015).

12. X. Zheng, J. Deotte, M. P. Alonso, G. R. Farquar, T. H. Weisgraber, S. Gemberling, H. Lee, N. X. Fang, C. M. Spadaccini, Design and optimization of a light-emitting diode projection micro-stereolithography three-dimensional manufacturing system. *Rev. Sci. Instrum.* **83**, 125001 (2012).

13. M. M. Emamia, F. Barazandeha, F. Yaghmaieba, Scanning-projection based stereolithography: Method and structure, *Sens. Actuat. A* **218**, 116–124 (2014).

14. X. Zheng, W. Smith, J. Jackson, B. Moran, H. Cui, D. Chen, J. Ye, N. X. Fang, N. Rodriguez, T. Weisgraber, C. M. Spadaccini. Multiscale metallic metamaterials. *Nat. Mater.* **15**, 1100–1106 (2016).

15. M. P. Lee, G. J. T. Cooper, T. Hinkley, G. M. Gibson, M. J. Padgett, L. Cronin, Development of a 3D printer using scanning projection stereolithography. *Sci. Rep.* **5**, 9875 (2015).

16. J. Kato, N. Takeyasu, Y. Adachi, H. Sun, S. Kawata, Multiple-spot parallel processing for laser micronanofabrication. *Appl. Phys. Lett.* **86**, 044102 (2005).

17. M. Shusteff, A. E. M. Browar, B. E. Kelly, J. Henriksson, T. H. Weisgraber, R. M. Panas, N. X. Fang, C. M. Spadaccini, One-step volumetric additive manufacturing of complex polymer structures. *Sci. Adv.* **3**, eaao5496 (2017).

18. B. E. Kelly, I. Bhattacharya, H. Heidari, M. Shusteff, C. M. Spadaccini, H. K. Taylor, Volumetric additive manufacturing via tomographic reconstruction. *Science* **363**, 1075–1079 (2019).

19. M. Regehly, Y. Garmshausen, M. Reuter, N. F. König, E. Israel, D. P. Kelly, C.-Y. Chou, K. Koch, B. Asfari, S. Hecht. Xolography for linear volumetric 3D printing. *Nature* **588**, 620–624 (2020).

20. M. Malinauskas, A. Žukauskas, S. Hasegawa, Y. Hayasaki, V. Mizeikis, R. Buividas, S. Juodkazis, Ultrafast laser processing of materials: From science to industry. *Light Sci. Appl.* **5**, e16133 (2016).

21. X. Zhou, Y. Peng, R. Peng, X. Zeng, Y. Zhang, T. Guo, Fabrication of large-scale microlens arrays based on screen printing for integral imaging 3D display. *ACS Appl. Mater. Interfaces* **8**, 24248–24255 (2016).

22. L. Stuerzebecher, T. Harzendorf, U. Vogler, U. D. Zeitner, R. Voelkel, Advanced mask aligner lithography: Fabrication of periodic patterns using pinhole array mask and Talbot effect. *Opt. Express* **18**, 19485–19494 (2010).

23. A. Kolodziejczyk, Z. Jaroszewicz, R. Henao, O. Quintero, The Talbot array illuminator: Imaging properties and a new interpretation. *J. Opt. A: Pure Appl. Opt.* **6**, 651–657 (2004).

24. M. Gonidec, M. M. Hamedi, A. Nemiroski, L. M. Rubio, C. Torres, G. M. Whitesides, Fabrication of nonperiodic metasurfaces by microlens projection lithography. *Nano Lett.***16**, 4125–4132 (2016).

25. R. Penrose, The role of aesthetics in pure and applied mathematical research. *Bull. Inst. Math. Appl.* **10**, 266–271 (1974).

26. L. Dal Negro, S. Boriskina, Deterministic aperiodic nanostructures for photonics and plasmonics applications. *Laser & Photon. Rev.* **6**, 178–218 (2012).

27. H. Tang, Z. Chen, N. Tang, S. Li, Y. Shen, Y. Peng, X. Zhu, J. Zang, Hollow-out patterning ultrathin acoustic metasurfaces for multifunctionalities using soft fiber/rigid bead networks. *Adv. Funct. Mater.* **28**, 1801127 (2018).

28. Z. Wu, Y. Zheng, Moiré chiral metamaterials. *Adv. Optical Mater.* **5**, 1700034 (2017).

29. M. Renner, G. von Freymann, Transverse mode localization in three-dimensional deterministic aperiodic structures. *Adv. Optical Mater.* **2**, 226–230 (2014).

30. E. Chen, Q. Yuan, Y.-P. Zhao, Topography-induced symmetry transition of droplets on quasi-periodically patterned surfaces. *Soft Matter* **14**, 6198–6205 (2018).

31. A. C. Hladky-Hennion, J. O. Vasseur, S. Degraeve, C. Granger, M. de Billy, Acoustic wave localization in one-dimensional Fibonacci phononic structures with mirror symmetry. *J. Appl. Phys.* **113**, 154901 (2013).

32. P. Wang, Y. Zheng, X. Chen, C. Huang, Y. V. Kartashov, L. Torner, V. V. Konotop, F. Ye, Localization and delocalization of light in photonic moiré lattices. *Nature* **577**, 42–46 (2020).

33. J.-H. Han, I. Kim, J.-W. Ryu, J. Kim, J.-H. Cho, G.-S. Yim, H.-S. Park, B. Min, M. Choi, Rotationally reconfigurable metamaterials based on moiré phenomenon. *Opt. Express* **23**, 17443–17449 (2015).

34. H. He, C. Qiu, L. Ye, X. Cai, X. Fan, M. Ke, F. Zhang, Z. Liu, Topological negative refraction of surface acoustic waves in a Weyl phononic crystal. *Nature* **560**, 61–64 (2018).

35. M. Thiel, G. von Freymann, M. Wegener, Layer-by-layer three-dimensional chiral photonic crystals. *Opt. Lett.* **32**, 2547–2549 (2007).

36. J. Geng, Three-dimensional display technologies. *Adv. Opt. Photon.* **5**, 456–535 (2013).

37. A. Manakov, J. F. Restrepo, O. Klehm, R. Hegedus, E. Eisemann, H.-P. Seidel, I. Ihrke, A reconfigurable camera add-on for high dynamic range, multispectral, polarization, and light-field imaging. *ACM Trans. Graph.* **32**, 4 (2013).

38. J. Arai, F. Okano, H. Hoshino, I. Yuyama, Gradient-index lens-array method based on real-time integral photography for three-dimensional images. *Appl. Optics* **37**, 2034–2045 (1998).

39. X. Xiao, B. Javidi, M. Martinez-Corral, A. Stern, Advances in three-dimensional integral imaging: Sensing, display, and applications [Invited]. *Appl. Optics* **52**, 546–560 (2013).

40. C. Parra-Cabrera, C. Achille, S. Kuhn, R. Ameloot, 3D printing in chemical engineering and catalytic technology: Structured catalysts, mixers and reactors. *Chem. Soc. Rev.* **47**, 209–230. (2018).

41. F. P. W. Melchels, K. Bertoldi, R. Gabbrielli, A. H. Velders, J. Feijen, D. W. Grijpma, Mathematically defined tissue engineering scaffold architectures prepared by stereolithography. *Biomaterials* **31**, 6909–6916 (2010).

42. W. Lee, D. Kang, J. Song, J. H. Moon, D. Kim, Controlled unusual stiffness of mechanical metamaterials. *Sci. Rep.* **6**, 20312 (2016).

43. N. Thomas, N. Sreedhar, O. Al-Ketan, R. Rowshan, R. K. A. Al-Rub, H. Arafat, 3D printed triply periodic minimal surfaces as spacers for enhanced heat and mass transfer in membrane distillation. *Desalination* **443**, 256–271 (2018).

44. R. He, S. Wang, G. Andrews, W. Shi, Y. Liu, Generation of customizable microwavy pattern through grayscale direct image lithography. *Sci. Rep.* **6**, 21621 (2016).

45. L. Li, A. Y. Yil, Microfabrication on a curved surface using 3D microlens array projection. *J. Micromech. Microeng.* **19**, 105010 (2009).

46. J. Tanida, T. Kumagai, K. Yamada, S. Miyatake, K. Ishida, T. Morimoto, N. Kondou, D. Miyazaki, Y. Ichioka, Thin observation module by bound optics (TOMBO): Concept and experimental verification. *Appl. Optics* **40**, 1806–1813 (2001).

47. H. Li, C. Guo, I. Muniraj, B. C. Schroeder, J. T. Sheridan, S. Jia, Volumetric light-field encryption at the microscopic scale. *Sci. Rep.* **7**, 40113 (2017).

48. M. Broxton, L. Grosenick, S. Yang, N. Cohen, A. Andalman, K. Deisseroth, M. Levoy, Wave optics theory and 3-D deconvolution for the light field microscope. *Opt. Express* **21**, 25418–25439 (2013).

49. TI DLP 4K Ultra High Definition (UHD) Display Chipset: www.ti.com/lit/ml/ssnb002/ssnb002.pdf

50. L. N. Kim, S. Choi, J. Kim, H. Kim, S. Kwon, Single exposure fabrication and manipulation of 3D hydrogel cell microcarriers. *Lab Chip* **11**, 48–51 (2011).

51. H. Kang, J. H. Park, D. Cho, A pixel based solidification model for projection based stereolithography technology. *Sens. Actuat. A* **178**, 223–229 (2012).

52. W. Yang, H. Yu, W. Liang, Y. Wang, L. Liu, Rapid fabrication of hydrogel microstructures using UV-induced projection printing. *Micromachines* **6**, 1903–1913 (2015).

53. G. Lazarev, A. Hermerschmidt, S. Krüger, S. Ostena, LCOS spatial light modulators: Trends and applications, in *Optical Imaging and Metrology: Advanced Technologies*, W. Osten, N. Reingand, Eds. (Wiley-VCH, 2012).

54. L. Zhang, F. Ou, W. C. Chong, Y. J. Chen, Q. M. Li, Wafer-scale monolithic hybrid integration of Si-based IC and III-V epi-layers—A mass manufacturable approach for active matrix micro-LED micro-displays. *J. Soc. Inf. Display* **26**, 137–145 (2018).

55. Epson 3LCD, 3-chip technology: https://epson.com/For-Home/Projectors/Pro-Cinema/PowerLite-Pro-Cinema-4855WU-WUXGA-3LCD-Projector-Kit/p/V11H543120MB

56. R. Wu, Z. Zheng, H. Li, X. Liu, Optimization design of irradiance array for LED uniform rectangular illumination. *Appl. Optics* **51**, 2257–2263 (2012).

57. A. Waldbaur, B. Waterkotte, K. Schmitz, B. E. Rapp, Maskless projection lithography for the fast and flexible generation of grayscale protein patterns. *Small* **8**, 1570–1578 (2012).

58. S. Surdo, R. Carzino, A. Diaspro, M. Duocastella, Single-shot laser additive manufacturing of high fill-factor microlens arrays. *Adv. Opt. Mater.* **6**, 1701190 (2018).

59. D. Zhang, Q. Xu, C. Fang, K. Wang, X. Wang, S. Zhuang, B. Dai, Fabrication of a microlens array with controlled curvature by thermally curving photosensitive gel film beneath microholes. *ACS Appl. Mater. Interfaces* **9**, 16604–16609 (2017).

60. G. Barbastathis, A. Ozcan, G. Situ, On the use of deep learning for computational imaging. *Optica* **6**, 921–943 (2019).

61. D. Wu, S.-Z. Wu, L.-G. Niu, Q.-D. Chen, R. Wang, J.-F. Song, H.-H. Fang, H.-B. Sun, High numerical aperture microlens arrays of close packing. *Appl. Phys. Lett.* **97**, 031109 (2010).

62. A. Orth, K. Crozier, Gigapixel fluorescence microscopy with a water immersion microlens array. *Opt. Express* **21**, 2361–2368 (2013).

63. M. Shusteff, "Volumetric additive manufacturing of polymer structures by holographically projected light fields", thesis, Massachusetts Institute of Technology, Cambridge, MA (2017).

64. Z. Xiong, H. Liu, R. Chen, J. Xu, Q. Li, J. Li, W. Zhang, Illumination uniformity improvement in digital micromirror device based scanning photolithography system. *Opt. Express* **26**, 18597–18607 (2018).

65. J. Kim, Y. Jeong, H. Kim, C.-K. Lee, B. Lee, J. Hong, Y. Kim, Y. Hong, S.-D. Lee, B. Lee, F-number matching method in light field microscopy using an elastic micro lens array. *Opt. Lett.* **41**, 2751–2754 (2016).

66. S.-I. Bae, K. Kim, S. Yang, K.-W. Jang, K.-H. Jeong, Multifocal microlens arrays using multilayer photolithography. *Opt. Express* **28**, 9082–9088 (2020).

67. H. Yabu, M. Shimomura, Simple fabrication of micro lens arrays. *Langmuir* **21**, 1709–1711 (2005).

68. G. C. L. Goff, J. Lee, A. Gupta, W. A. Hill, P. S. Doyle, High-throughput contact flow lithography. *Adv. Sci.* **2**, 1500149 (2015).

69. H. Zhang, F. Yang, J. Dong, L. Du, C. Wang, J. Zhang, C. F. Guo, Q. Liu, Kaleidoscopic imaging patterns of complex structures fabricated by laser-induced deformation. *Nat. Commun.* **7**, 13743 (2016).

70. S. Kim, D. H. Kim, W. Kim, Y. T. Cho, N. X. Fang, Additive manufacturing of functional microarchitected reactors for energy, environmental, and biological applications. *Int. J. of Precis. Eng. and Manuf.-Green Tech.* **8**, 303–326 (2021).

71. E. Behroodi, H. Latifi, F. Najafi, A compact LED-based projection microstereolithography for producing 3D microstructures. *Sci. Rep.* **9**, 19692 (2019).

72. C.-C. Sun, W.-T. Chien, I. Moreno, C.-T. Hsieh, M.-C. Lin, S.-L. Hsiao, X.-H. Lee, Calculating model of light transmission efficiency of diffusers attached to a lighting cavity. *Opt. Express* **18**, 6137–6148 (2010).

73. I. Moreno, M. Avendaño-Alejo, R. I. Tzonchev, Designing light-emitting diode arrays for uniform near-field irradiance. *Appl. Optics* **45**, 2265–2272 (2006).

74. H. Chen, C. Liou, S. Siao, Illumination distribution and signal transmission for indoor visible light communication with different light-emitting diode arrays and pre-equality circuits. *Opt. Eng.* **54**, 115106 (2015).

75. L. Han, G. Mapili, S. Chen, K. Roy, Projection microfabrication of three-dimensional scaffolds for tissue engineering. *J. Manuf. Sci. Eng.* **130**, 021005 (2008).

76. C. Xia, N. X. Fang, 3D micro fabricated bioreactor with capillaries. *Biomed. Microdevices* **11**, 1309–1315 (2009).

77. J. Choi, R. Wicker, S. Lee, K. Choi, C. Ha, I. Chung, Fabrication of 3D biocompatible/biodegradable micro-scaffolds using dynamic mask projection microstereolithography. *J. Mater. Process. Technol.* **209**, 5494–5503 (2009).

78. J. Choi, R. B. Wicker, S. Cho, C. Ha, S. Lee, Cure depth control for complex 3D microstructure fabrication in dynamic mask projection microstereolithography. *Rapid Prototyp. J.* **15**, 59–70 (2009).

79. C. Zhou, Y. Chen, Z. Yang, B. Khoshnevis, Digital material fabrication using mask-image-projection-based stereolithography. *Rapid Prototyp. J.* **19**, 153–165 (2013).

80. A. R. Schultz, P. M. Lambert, N. A. Chartrain, D. M. Ruohoniemi, Z. Zhang, C. Jangu, M. Zhang, C. B. Williams, T. E. Long, 3D printing phosphonium ionic liquid networks with mask projection microstereolithography. *ACS Macro Lett.* **3**, 1205–1209 (2014).

81. K. Kim, W. Zhu, X. Qu, C. Aaronson, W. R. McCall, S. Chen, D. J. Sirbuly, 3D optical printing of piezoelectric nanoparticle–polymer composite materials. *ACS Nano* **8**, 9799–9806 (2014).

82. A. P. Zhang, X. Qu, P. Soman, K. C. Hribar, J. W. Lee, S. Chen, S. He, Rapid fabrication of complex 3D extracellular microenvironments by dynamic optical projection stereolithography. *Adv. Mater.* **24**, 4266–4270 (2012).

83. P. Soman, B. T. D. Tobe, J. W. Lee, A. M. Winquist, I. Singec, K. S. Vecchio, E. Y. Snyder, S. Chen, Three-dimensional scaffolding to investigate neuronal derivatives of human embryonic stem cells, *Biomed. Microdevices* **14**, 829–838 (2012).

84. Y. L. Jeyachandran, N. Meyerbröker, A. Terfort, M. Zharnikov, Maskless ultraviolet projection lithography with a biorepelling monomolecular resist. *J. Phys. Chem. C* **119**, 494–501 (2015).

85. Lawrence Livermore National Laboratory (LLNL), "Large-area projection micro-stereolithography (LAPμSL)" (Publication LLNL-MI-670797, LLNL, 2015; https://ipo.llnl.gov/sites/default/files/2019-09/lapusl.pdf).

86. M. Shusteff, "Projection microstereolithography for architected materials" (Tech. Rep. LLNL-TR-666552, Lawrence Livermore National Laboratory (LLNL), 2015).

87. R. Janusziewicz, J. R. Tumbleston, A. L. Quintanilla, S. J. Mecham, J. M. DeSimone, Layerless fabrication with continuous liquid interface production. *Proc. Natl. Acad. Sci. U.S.A.* **113**, 11703–11708 (2016).

88. H. Gong, M. Beauchamp, S. Perry, A. T. Woolleyb, G. P. Nordin, Optical approach to resin formulation for 3D printed microfluidics. *RSC Adv.* **5**, 106621–106632 (2015).

89. A. S. Limaye, D. W. Rosen, Process planning method for mask projection micro-stereolithography. *Rapid Prototyp. J.* **13**, 76–84 (2007).

90. Y. Jung, H. Lee, T. Park, S. Kim, S. Kwon, Programmable gradational micropatterning of functional materials using maskless lithography controlling absorption. *Sci. Rep.* **5**, 15629 (2015).

91. F. Zhou, W. Cao, B. Dong, T. Reissman, W. Zhang, C. Sun, Additive manufacturing of a 3D terahertz gradient-refractive index lens. *Adv. Optical Mater.* **4**, 1034–1040 (2016).

92. F. Zhou, Y. Bao, W. Cao, C. T. Stuart, J. Gu, W. Zhang, C. Sun, Hiding a realistic object using a broadband terahertz invisibility cloak. *Sci. Rep.* **1**, 78 (2011).

93. A. Urrios, C. Parra-Cabrera, N. Bhattacharjee, A. M. Gonzalez-Suarez, L. G. Rigat-Brugarolas, U. Nallapatti, J. Samitier, C. A. DeForest, F. Posas, J. L. Garcia-Corderob, A. Folch, 3D-printing of transparent bio-microfluidic devices in PEG-DA, *Lab Chip* **16**, 2287–2294 (2016).

94. Q. Ge, A. H. Sakhaei, H. Lee, C. K. Dunn, N. X. Fang, M. L. Dunn. Multimaterial 4D printing with tailorable shape memory polymers. *Sci. Rep.* **6**, 31110 (2016).

95. J. Na, N. P. Bende, J. Bae, C. D. Santangelob, R. C. Hayward, Grayscale gel lithography for programmed buckling of non-Euclidean hydrogel plates, *Soft Matter* **12**, 4985–4990 (2016).

96. N. P. Macdonald, J. M. Cabot, P. Smejkal, R. M. Guijt, B. Paull, M. C. Breadmore, Comparing microfluidic performance of three-dimensional (3D) printing platforms, *Anal. Chem.* **89**, 3858–3866 (2017).
